# A Real-Time Polyp-Detection System with Clinical Application in Colonoscopy Using Deep Convolutional Neural Networks

**DOI:** 10.3390/jimaging9020026

**Published:** 2023-01-24

**Authors:** Adrian Krenzer, Michael Banck, Kevin Makowski, Amar Hekalo, Daniel Fitting, Joel Troya, Boban Sudarevic, Wolfgang G. Zoller, Alexander Hann, Frank Puppe

**Affiliations:** 1 Department of Artificial Intelligence and Knowledge Systems, Julius-Maximilians University of Würzburg, Sanderring 2, 97070 Würzburg, Germany; 2 Interventional and Experimental Endoscopy (InExEn), Department of Internal Medicine II, University Hospital Würzburg, Oberdürrbacher Straße 6, 97080 Würzburg, Germany; 3 Department of Internal Medicine and Gastroenterology, Katharinenhospital, Kriegsbergstrasse 60, 70174 Stuttgart, Germany

**Keywords:** machine learning, deep learning, endoscopy, gastroenterology, automation, object detection, video object detection, real-time

## Abstract

Colorectal cancer (CRC) is a leading cause of cancer-related deaths worldwide. The best method to prevent CRC is with a colonoscopy. During this procedure, the gastroenterologist searches for polyps. However, there is a potential risk of polyps being missed by the gastroenterologist. Automated detection of polyps helps to assist the gastroenterologist during a colonoscopy. There are already publications examining the problem of polyp detection in the literature. Nevertheless, most of these systems are only used in the research context and are not implemented for clinical application. Therefore, we introduce the first fully open-source automated polyp-detection system scoring best on current benchmark data and implementing it ready for clinical application. To create the polyp-detection system (ENDOMIND-Advanced), we combined our own collected data from different hospitals and practices in Germany with open-source datasets to create a dataset with over 500,000 annotated images. ENDOMIND-Advanced leverages a post-processing technique based on video detection to work in real-time with a stream of images. It is integrated into a prototype ready for application in clinical interventions. We achieve better performance compared to the best system in the literature and score a F1-score of 90.24% on the open-source CVC-VideoClinicDB benchmark.

## 1. Introduction

Colorectal cancer (CRC) is the second leading cause of cancer-related deaths worldwide [1]. One of the best methods to avoid CRC is to perform a colonoscopy to detect the potential disease as early as possible. A colonoscopy examines the large intestine (colon) with a long flexible tube inserted into the rectum. A small camera is mounted at the end of the tube, enabling the physician to look inside the colon [2]. During this procedure, the colonoscopist searches for polyps and examines them closely. Polyps are protrusions of the mucosal surface of various shapes and sizes that can be benign or malignant and, thus, can develop into CRC. Polyps grow on the colon lining, which often does not cause symptoms. The two main types are non-neoplastic and neoplastic polyps. Non-neoplastic polyps usually do not become cancerous and polyps of type neoplastic might become cancerous [2]. Even if many polyps are not cancerous, some become colon cancer. Ideally, the colonoscopist detects every polyp during a colonoscopy and decides, on closer inspection, whether it needs to be removed. Nevertheless, there is still a potential risk of polyps being missed. It has been shown that up to 27% of diminutive polyps are overlooked by physicians [3,4], which happens due to lack of experience or fatigue. It has also been shown that even a general error rate of 20–24% leads to a high risk of patients dying from CRC [5,6]. Two studies have shown that the missing rate is related to the size of the polyp. Kim et al., showed that polyps of size ≤5 mm, 5–10 mm and ≥10 mm had a missing rate of 35.4%, 18.8%, and 4.9%, respectively [7]. Ahn et al., demonstrated missing rates of 22.9%, 7.2%, and 5.8% for sizes of ≤5 mm, 5–10 mm, and ≥10 mm, respectively [8]. Both studies also found that the missing rate was higher when the patient had more than one polyp. Additionally, a systematic review calculated a similar value and received missing rates of 2%, 13%, and 26% for polyp sizes of ≥10 mm, 5–10 mm, and 1–5 mm, respectively [6]. This indicates that smaller polyps have a higher risk of being missed by the colonoscopist. Missed polyps can have fatal consequences for the patient. Thus, the colonoscopist must detect and remove all potential cancerous polyps to minimize the risk of CRC [8].

To avoid missing polyps, computer science research methods have been developed to assist physicians during the colonoscopy. The use of computers to detect polyps is called *computer-aided detection (CAD)*. The research field already has publications examining the problem of polyp detection. Nevertheless, most of these systems are only used in research context and are not developed to be ready for clinical application. There are commercial systems ready for clinical application; however, they are very expensive [9,10,11,12]. Therefore, we introduce the first fully open-source system scoring best on current benchmark data and implementing it for clinical-ready applications.

The main contributions of our paper are:

(1) *We introduce the first fully open-source (https://fex.ukw.de/public/download-shares/d8NVHA2noCiv7hXffGPDEaRfjG4vf0Tg, accessed on 18 December 2022), clinically ready, real-time polyp-detection system.*
(2) *We show that the system outperforms current systems on benchmark data with real-time performance.*
(3) *We introduce a novel post-processing method working in real-time based on REPP [13] and use a new metric for polyp detection, which has value for clinical usage.*

**Figure 1:**
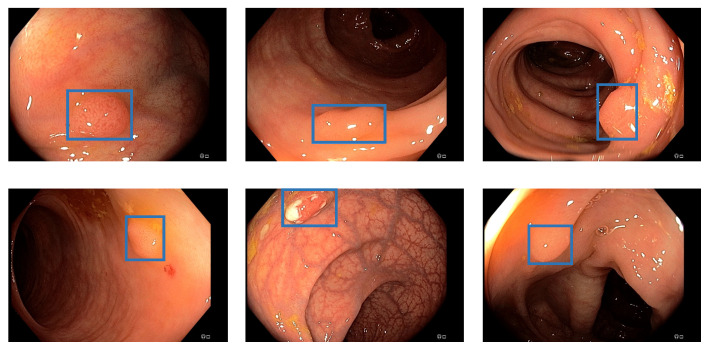
Detection examples. This figure illustrated some detection examples of the polyp-detection system on our own data (EndoData).

Additionally, the polyp-detection system was publicly funded and developed by computer engineers and endoscopists in the same workgroup to ensure high-quality polyp detection. Figure 1 shows the results of the polyp-detection system. To overview existing work and properly allocate our paper in the literature, we describe a brief history from general polyp detection with handcrafted features to state-of-the-art polyp detection with deep learning techniques.

### 1.1. A Brief History of Computer-Aided Polyp Detection

The field of CAD is divided into two subfields, CADe and CADx. CADe deals with the detection and localization of polyps and CADx deals with the characterization of polyps. This paper will focus exclusively on the CADe area. This section only considers methods that localize polyps by specifying a rectangular section of the screen, a *bounding box*.

#### 1.1.1. Computer-Aided Detection with Handcrafted Features

The first approaches for computer-aided detection of polyps were explored as early as the late 1990s. For example, Krishnan et al., proposed using curvature analysis to detect polyps by shape [14]. Another method was presented in 2003 by Karkanis et al. They used wavelet transform to detect polyps based on their color and texture [15]. Hwang et al., used a new technique to distinguish the elliptical shape features of polyp regions from non-polyp regions. They compared the features based on curvature, intensity, curve direction, and distance from the edge [16]. Bernal et al. (2012) proposed another method by converting images of polyps to grayscale so that the elevations of the polyps could be seen. Subsequently, the authors illuminated the outlines of the polyps, which they termed valleys. Based on the intensity of the valleys, the polyps were extracted and localized [17].

Furthermore, expert knowledge was used to handcraft rules for detecting polyps based on specific properties, such as size, shape, and color. Newer examples can be found in [18,19], both of which use support vector machines (SVMs). Additionally, real-time detection with handcrafted features was tested in clinical applications [20]. The authors used a weighted combination of color, structure, textures, and motion information to detect image areas where a polyp is possibly located. The detection rate was 73%. Nevertheless, the rise of convolutional neural network (CNN)-based methods in image processing has superseded all of these techniques, as CNN methods have proven to show better results.

#### 1.1.2. Methods Involving CNNs

Computer-aided polyp recognition was particularly shaped by various deep learning methods from the beginning of the last decade. We listed an overview of the essential models on still image datasets in Table 1. Specifically, a great deal of research interest has developed in the object recognition capabilities of CNNs. For example, in 2015, authors Zhu et al., presented a seven-layer CNN as a feature extractor with a SVM as a classifier to detect anomalies in endoscopy images [21]. The system was trained on custom data. The earlier approaches considered using an existing CNN architecture to localize polyps, the AlexNet [22,23,24]. This was developed for general image classification, i.e., not specifically for the medical field. The paper by Tajbakhsh et al. [23] states that the AlexNet [22] for polyp detection is better not trained thoroughly, i.e., starting from random weights, but the already pre-trained weights should be used. It is shown that *transfer learning* is a practical approach in the presence of limited data, as generally given in the medical field.

Yuan et al. [24] first extract an attractive image section via edge-finding algorithms as a preliminary step and use it as input to the AlexNet [22]. This resulted in a high recall of 91.76% compared to the state-of-the-art at that time. Mo et al. [31] are the first to use the unmodified *faster region-based convolutional neural network* (Faster R-CNN) [32] architecture for polyp detection. This allows the detection of polyps that are mostly obscured or very close to the camera, unlike previous models. The model is trained on the CVC-ClinicDB data. The model is robust to illumination changes or circular bubbles, but it misses some smaller polyps and sometimes becomes too guided by an oval shape, increasing the number of false positives (FP). The authors also wanted to focus on these problems in the future. Shin et al. [33] were the first to use the inception-residual neural network (ResNet) [34] architecture unmodified for polyp detection. The model is trained on the ASU-Mayo-Video-DB. They also added two post-processing methods, *false positive learning* and *offline learning*, to further improve the model’s performance. The advantage of the model was that the entire frame could be used for training rather than a previous patch extraction step. As with Mo et al., one problem with the model is the high number of FP triggered by polyp-like structures. The authors plan to focus on improving the speed in the future, which was only 2.5 frames per second (FPS). Zheng et al. [30] use the unmodified you only look once (YOLO) architecture [35]. Again, the advantages are that there is only one processing step, so there is no preliminary step to extract an regions of interest (RoI). As a result, the model was faster than the two-step methods but only achieved 16 FPS. Further, the authors note that the CNN features of *white light* and *narrow-band* images differed greatly, thus, they should be considered separately. The model is trained on the CVC-CLinicDB, CVC-ColonDB, and custom data. Liu et al. [28] implemented and compared different back-end models as feature extractors for the *single shot detection* architecture (SSD) [36]. These were *ResNet50* [37], *Visual Geometry Group-16 (VGG-16)* [38], and *InceptionV3* [39], with InceptionV3 showing the best balanced result. The advantages of the models are robustness to size and shape, as well as speed, which is real-time capable at 30 FPS. The models are trained on the CVC-CLinicDB, CVC-ColonDB, and ETIS-Larib data. In the future, other back-end models could result in a further boost in performance.

Zhang et al. [40] used the *SSD-GPNet*, which is based on the SSD architecture [36], but tries to incorporate information that is normally lost by the standard pooling layers into the result through various customized pooling methods. Since it is based on the SSD architecture, this method is also fast and achieves real-time capability at 50 FPS; it also achieves good recall, especially for small polyps. In the future, the authors want to test their approaches for other diseases and find more ways to use as much image information as possible without increasing the complexity of the models. Furthermore, Zhang et al., presented another deep learning method for polyp detection and localization. They presented a special single-shot multi-box detector-based CNN model that reused displaced information through max-pooling layers to achieve higher accuracy. At 50 FPS, the method provided real-time polyp detection while achieving a mean average precision of up to 90.4% [40]. The model is trained on custom data. Authors Bagheri et al., staged a different idea in which they first converted the input images into three color channels and then passed them to the neural network. This allows the network to learn correlated information using the preprocessed information about the color channels to locate and segment polyps [41]. With the same goal, Sornapudi et al., in their paper, used region-based CNNs to localize polyps in colonoscopy images and in wireless capsule endoscopy (WCE) images. During localization, images were segmented and detected based on polyp-like pixels [42].

In addition to CNNs, research is also being conducted on other deep learning methods for polyp detection. For example, a special sparse autoencoder method called stacked sparse autoencoder with image manifold constraint was used by Yuan and Meng [43] to detect polyps in WCE images. A sparse autoencoder is an artificial neural network commonly used for unsupervised learning methods [44]. The sparse autoencoder achieved 98% accuracy in polyp detection [43]. The system is trained and tested on the ASU-Mayo-Video-DB. Wang et al. [27] used the *AFP-Net*. Unlike an SSD model, an AFP-Net model does not require predefined anchor boxes, it is *anchor free*. It was the first application of such an architecture for polyp detection. Through *context enhancement module* (CEM), a *cosine ground-truth projection* and a customized loss function, the speed was increased and 52.6 FPS was achieved, which is real-time capability. In the future, the authors still want to improve the detection of hard-to-detect small and flat polyps. The model is trained on the CVC-ClinicVideoDB. Liu et al. [26] used an *anomaly detection generative adversarial network* (ADGAN), which is based on the Wasserstein GAN [45]. ADGAN aims to learn only based on healthy images without polyps to reconstruct them. If this model receives an image with a polyp as input, the model cannot reconstruct it, so at this point in the output, there is a noticeably large difference from the input, which is easy to check. The problem of connecting the input to the latency space of the GAN was solved by a second GAN. In addition, a new loss function was added to improve performance even further. The model is trained on custom data.

The advantage of this approach is that no costly annotated datasets are needed and significantly larger amounts of data of normal intestinal mucosa are available. For the future, the authors want to ensure that frames with biological disturbances, such as stool residue or water, are also processed well since these are often sorted out beforehand from many datasets. Yuan et al. [25] use the *DenseNet-UDCS* architecture for frame classification, not localization, of WCE images. The DenseNets [46] structure is kept unchanged, but the loss function is adapted. On the one hand, weighting is introduced to compensate for the significant imbalance in the size of the classes (with or without polyps). On the other hand, the loss function is adapted to be class sensitive. It forces that similar features are learned for the same class and the features of the other class have as significant differences as possible. These adaptations improve performance and can be easily applied to other applications and models. In the future, the researchers still want to find a way to compensate for different illuminations by pre-processing and testing attention-based methods. Another method is to use transformers in combination with CNNs. Zhang et al., used in parallel the ability to view global information of the whole image through the attention layers of the transformers and the detection of the CNNs to efficiently segment polyps. In addition, a new fusion technique called BiFusion was used to fuse the features obtained by the transformers and the CNNs. The resulting method called TransFuse stood out mainly because of its segmentation time of 98.7 FPS [47]. The model is trained on custom data.

Furthermore, Jha et al. [48] proposed a segmentation, detection, and classification model. The model achieves a mean average precision (mAP) of 81.66 while being very fast, with 180 FPS on an NVIDIA Volta 100 GPU. The results are evaluated on the Kvasir-SEG dataset. Another approach is combining different networks in an ensemble to increase the polyp-detection performance further. While ensemble techniques significantly increase detection systems’ performance, the downside is that those systems mostly have high latency. For example, if an ensemble combined five different neural networks, the computational complexity would be increased at least five times. Therefore, ensemble techniques are not implemented for real-time detection. The paper of [49] shows a polyp-detection system using an ensemble. The authors combined three different models by using majority voting to increase the performance of the polyp-detection system.

Additionally, Livovsky et al. [50] trained a large-scale AI called detection of elusive polyps (DEEP2). They used 3611 h of colonoscopy videos for training and 1393 h for testing. They trained and evaluated their AI on a custom dataset. As neural network architecture they used RetinaNet. Livovsky et al., achieved a recall of 88.5% with polyps visible longer than 5 s. Moreover, they showed recall and specificity results for different lengths of polyps visibility.

#### 1.1.3. 3D and Temporal Methods

While older publications are evaluated on still-image benchmarks, such as the CVC-ClinicDB, the new state-of-the-art is evaluated on the more challenging and more realistic video dataset, such as CVC- VideoClinicDB. For example, Wang et al. [27] have a high score of 97.88% on the CVC-ClinicDB dataset. Nevertheless, this dataset only involves 612 still images. We reconstructed the algorithm of Wang et al. [27] but could not reproduce the results on the video datasets. For these video datasets, all frames are extracted and polyps in these frames are annotated with corresponding bounding boxes. We listed an overview of the essential models on video datasets in Table 2. Another approach is to use temporal information within the video. In the methods mentioned above, only single frames are considered. Thus, information that is given by the sequence of the frames is lost. In Itoh et al. [51], temporal information is included through a *3D-ResNet*. In addition, a weighted loss function and selection of so-called *hard negative frames* address the problem of training-data class imbalance. These lead to an improvement of 2% F1-score. However, one problem is that the model has a higher probability of being overfitted than its 2D counterpart because it has more parameters and is not applicable in real-time. Zhang et al. [29] combine the output of a conventional SSD model [36] via a *Fusion module* with a generated *optical flow*. This is similar to a heat map showing motion over short periods and is easy to compute. This approach is much less complex and, therefore, faster than other temporal systems that use 3D methods; still, it is not applicable for real-time polyp detection. Misawa et al. [52] use a *3D-CNN* to include temporal information. This allows many different types of polyps to be well detected. The model is trained on custom data.

Additionally, Qadir et al. [55] use a conventional localization model, such as SSD [36], or Faster R-CNN [32], and further process the output of these through a *False Positive Reduction Unit*. This looks at the position of the generated bounding boxes over the seven preceding and following frames and tries to find and possibly correct outliers. Because future frames are used, there is a small delay, but the actual calculation of the *False Positive Reduction Unit* is fast. A different and promising method was provided by Qadir et al., in a two-step process. They used a CNN in the first step, which generated several RoIs for classification. Then, these proposed RoIs were compared based on the subsequent frames and their RoIs and classified into true positive (TP) and false positive (FP). This method assumes that the frame in a video should be similar to the next frame. It intends to reduce the percentage of false predictions [55]. Because CNNs are sensitive to noise in the data, they may produce a high count of FPs. Another approach is therefore using a two-stage method that first suggests multiple RoIs. Then, the current proposed RoIs are categorized as TPs and FPs by considering the RoIs of the following frames [55]. With this method, they are reducing the number of FPs and reaching state-of-the-art results. The model is trained on the ASU-Mayo-Video-DB and custom data.

Furthermore, Misawa et al., developed a real-time polyp-detection system based on YOLOv3. The system has a speed of 30 FPS and achieves a F1-score of 97.05% on their open-source dataset (SUN-Colonoscopy) [11]. Moreover, Xu et al. [54] designed a 2D CNN detector including spatiotemporal information involving a structural similarity (SSIM) to advance polyp detection further while maintaining real-time speed. The model is trained on custom data. In 2022 Nogueira et al., published a real-time polyp-detection system using the YOLOv3 model with an object-tracking algorithm. The system scores a F1-score of 88.10% on their custom dataset.

## 2. Materials and Methods

This section explains the software and hardware for our polyp-detection system. We call our polyp-detection system ENDOMIND-Advanced. An early, preliminary version of our detection system was experimentally tested and called ENDOMIND [56]. Nevertheless, ENDOMIND used an early version of YOLOv5 that did not involve our preprocessing, hyperparameter, optimization, and post-processing, and was trained with a smaller dataset. First, we introduce our datasets for training and testing the AI. Afterward, we illustrate typical challenges in the field of automatic polyp detection. We continue by showing our data preprocessing and data augmentation. We then show the full polyp-detection system and explain its components. The full polyp-detection system involves the CNN-based YOLOv5 model and our implemented post-processing solution real-time REPP (RT-REPP), which uses an algorithm called *Robust and Efficient Post-Processing* (REPP) [13]. We close this section by elaborating on the clinical application of our system.

### 2.1. Datasets

**Figure 2:**
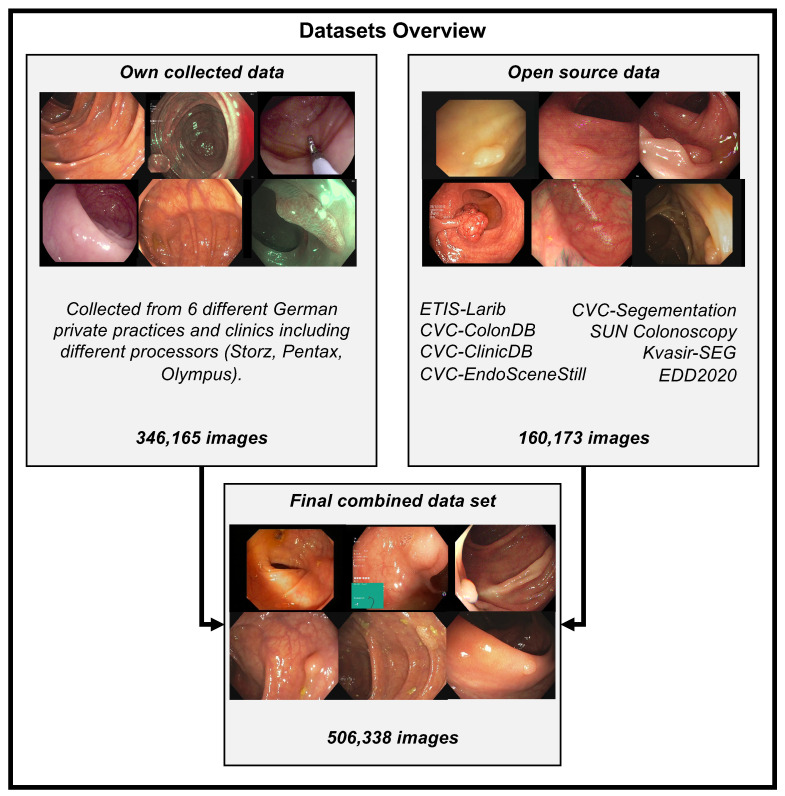
Training datasets overview. This figure illustrates all the data we combined and gathered for training the polyp-detection system. Open-source data are combined with our data collected from different German private practices to create one dataset with 506,338 images. Storz, Pentax, and Olympus are different endoscope manufacturing companies, and we collected the data using their endoscope processors. The different open source datasets have the following number of images: ETIS-Larib: 196, CVC-Segmentation: 56, SUN Colonoscopy: 157,882, Kvasir-SEG: 1000, EDD2020: 127, CVC-EndoSceneStill: consist of CVC-ColonDB: 300 and CVC-ClinicDB: 612. Overall this sums up to 160,173 open-source images.

Obtaining qualitative data on an appropriate scale is often one of the biggest problems for applying deep learning methods. This is no different for colonoscopy videos/images for polyp detection. The difficulties in the acquisition are due to data protection issues on the one hand and the expensive and time-consuming but necessary annotation of the data by experienced medical experts. Therefore, for developing our model, we use our own data and all the publicly available data we could find on the internet and in the literature. For training our model, we combined the available online sources and our own data to forge a dataset of 506,338 images. Figure 2 shows an overview of the data material. The details about creating our own dataset will follow below. All data consist of images and bounding box coordinates of boxes referring to the image. For a listing of publicly available datasets we used for training, we show the following overview:

- ETIS-Larib [57] 2014: It contains 196 polyp images from 34 different videos and shows 44 different polyps. ETIS-LaribPolypDB [57] is from the *MICCAI 2015 Endoscopic Vision Challenge* and was used as the testing dataset in the challenge. Here, we include this dataset in our training dataset. It has 196 polyp images with the corresponding mask for boxes. For our training, we extracted the bounding boxes from the segmentation masks. The size of the images is 348 × 288 pixels. This dataset contains no healthy mucosa images. This dataset contains no healthy mucosa images. The data are available on request in the CVC-Colon repository (http://www.cvc.uab.es/CVC-Colon/index.php/databases/, accessed on 18 December 2022).
- CVC-VideoClinicDB [58] 2017: The CVC-VideoClinicDB [59] dataset was provided in the context of the GIANA sub-challenge that was part of the *MICCAI 2017 Endoscopic Vision Challenge*. This dataset contains 18,733 frames from 18 videos without ground truth and 11,954 frames from 18 videos with ground truth. We exclusively used these frames for final evaluation. It has to be noted that the ground truth masks that label a polyp are approximated by using ellipses. Furthermore, we filtered out all images with no polyps (empty mask) and only used frames with at least one polyp for training. The size of the images is 574 × 500 pixels. This dataset is only used for testing in this paper. The data are available upon request in the CVC-Colon repository (http://www.cvc.uab.es/CVC-Colon/index.php/databases/, accessed on 18 December 2022).
- CVC-EndoSceneStill [60] 2017: It combines *CVC-ColonDB* and *CVC-ClinicDB* and contains 912 polyp images from 44 videos of 36 patients. CVC-EndoSceneStill [60] is a dataset that combines CVC-ColonDB [17] (CVC-300) and CVC-Clinic-DB [58,61] (CVC-612). Both datasets gave each image a border, specular, lumen, and segmentation mask. The border mask marks the black border around each image, the specular mask indicates the reflections that come from the endoscope light, and the lumen mask labels the intestinal lumen, which is the space within an intestine. The segmentation mask contains polyp markings that tag visible polyps within a picture. Because we needed the bounding box from a polyp, we only used the segmentation masks and extracted a bounding box by calculating a box that fits around a single blob. The dataset CVC-ColonDB [17,60] contains 300 selected images from 13 polyp video sequences with a resolution of 574 × 500 and CVC-Clinic-DB [58,60,61] holds 612 images from 31 polyp video sequences with a size of 348 × 288 pixels. This dataset contains no healthy mucosa images. The data are available on request in the CVC-Colon repository (http://www.cvc.uab.es/CVC-Colon/index.php/databases/, accessed on 18 December 2022).
- Kvasir-SEG [62] 2020: The dataset contains 1000 polyp images with corresponding 1071 masks and bounding boxes. Dimensions range from 332 × 487 to 1920 × 1072 pixels. Gastroenterologists verified the images from *Vestre Viken Health Trust* in Norway. Most images have general information displayed on the left side and some have a black box in the lower left corner, which covers information from the endoscope position marking probe created by ScopeGuide (Olympus). This dataset contains no healthy mucosa images. The data are available in the Kvasir-SEG repository (https://datasets.simula.no/kvasir-seg/, accessed on 18 December 2022).
- SUN Colonoscopy Video Database [11] 2021: The database was developed by Mori Laboratory, Graduate School of Informatics, Nagoya University. It contains 49,136 fully annotated polyp frames taken from 100 different polyps. These images were collected at the Showa University Northern Yokohama and annotated by expert endoscopists at Showa University. Additionally, 109,554 non-polyp frames are included. The size of the images is 1240 × 1080 pixels. The data are available in the SUN Colonoscopy Video repository (http://sundatabase.org/, accessed on 18 December 2022).
- CVC-Segementation-HD [60] 2017: This dataset was made available within the GIANA Polyp Segmentation sub-challenge that was part of the *MICCAI 2017 Endoscopic Vision Challenge*. It contains 56 high-resolution images with a size of 1920 × 1080 pixels. This dataset contains no healthy mucosa images. There is a binary mask from which we have extracted the bounding boxes for each image. The data are available on request in the CVC-Colon repository (http://www.cvc.uab.es/CVC-Colon/index.php/databases/, accessed on 18 December 2022).
- Endoscopy Disease Detection Challenge 2020 (EDD2020) [63]: The EDD2020 challenge released a dataset containing five different classes with masks and bounding boxes for each image and polyp instance. We extracted all images labeled as a polyp and stored the relevant bounding boxes into a custom JSON file for our task. These data contain 127 images, and the size of the images is 720 × 576 pixels. This dataset contains no healthy mucosa images. The data are available on request in the ENDOCV repository (https://endocv2022.grand-challenge.org/Data/, accessed on 18 December 2022).

#### Own Data Creation

Previously, we designed a framework that utilizes a two-step process involving a small expert annotation part and a large non-expert annotation part [64]. This shifts most of the workload from the expert to a non-expert while still maintaining proficient high-quality data. Both tasks are combined with artificial intelligence (AI) to enhance the annotation process efficiency further. Therefore, we used the software Fast Colonoscopy Annotation Tool (FastCat) to handle the entirety of this annotation process. This tool assists in the annotation process in endoscopic videos. The design of this tool lets us label coloscopic videos 20 times faster than traditional labeling. The annotation process is split between at least two people. At first, an expert reviews the video and annotates a few video frames to verify the object’s annotations. In the second step, a non-expert has visual confirmation of the given object and can annotate all following and preceding images with AI assistance. To annotate individual frames, all frames of the video must be extracted. Relevant scenes can be pre-selected by an automated system, and this prevents the expert from reviewing the entire video every single time. After the expert has finished, relevant frames will be selected and passed on to an AI model. This allows the AI model to detect and mark the desired object on all following and preceding frames with an annotation. The non-expert can adjust and modify the AI predictions and export the results, which can then be used to train the AI model. Furthermore, the expert annotates the Paris classification [65], the size of the polyp, its location, the start and end frame of the polyp, and one box for the non-expert annotators.

We built a team of advanced gastroenterologists and medical assistants. We created a dataset of 506,338 images, including the open-source images listed above. Figure 2 shows an overview of the different datasets. Our dataset consists of 361 polyp sequences and 312 non-polyp sequences. The polyp sequence was selected in high quality as we were generally only annotating the first 1–3 s of the polyp’s appearance, which is critical for detecting polyps in a real clinical scenario. We combined training material from six centers involving three different endoscope manufacturers, named Karl Storz GmbH und Co. KG (Storz), Ricoh Company Ltd. (Pentax), and Olympus K.K. (Olympus). Overall, 91% of the images are from Olympus, 5% are from Pentax, and 4% are from Storz processors. We create a dataset of 24 polyp sequences involving 12,161 images and 24 non-polyp sequences involving 10,695 images for the test data (EndoData). Therefore, the test data consist of an additional 22,856 images. We assured the independency of the test data as EndoData is created from a different clinic with different polyps and patients compared to the training data.

### 2.2. Current Challenges

There are still some challenges left. The most important of these can be divided into two categories: on the one hand, the hurdles to achieving the actual goal, real-time support of physicians, and on the other hand, problems arising from the acquisition or use of the datasets. The volume and quality of the data is a constant problem factor, and although there are various ways to deal with these problems, they still need to be solved.

#### 2.2.1. Training and Testing Datasets

The biggest problem faced by most papers, e.g., [66] or [67], is the low availability of usable datasets. This refers not only to the number and size of datasets but also to the usually significant imbalance between the two classes *healthy frames* and *frames with polyps*. The need for more availability of pathological data is a common problem for medical deep learning applications. However, there are also various attempts to deal with it.

One widely used approach by Kang et al. [66] is *transfer learning*, which uses a model that has already been pre-trained on a non-medical dataset and is re-trained with a polyp dataset. The advantage is that sufficiently large non-medical datasets are readily available. With these, general problem-independent skills, such as edge detection, can already be learned well and only need to be fine-tuned to the specialized application.

Another method that almost all papers use is *data augmentation* of the training data. This involves slightly modifying the existing training data using various methods, thus increasing the available data and the system’s robustness to the applied transformations. Examples of such transformations are rotation, mirroring, blur, and color adjustments [25].

An interesting approach by Guo et al. [68] is that the test data are also augmented at test time. More precisely, they are rotated and then passed to the model. To arrive at the result for the original image, all generated masks are rotated back accordingly and averaged. This makes the system more robust against rotation and leads to better accuracy.

Other ideas can be found in Thomaz et al. [69], where a CNN inserts polyps into healthy images to increase the available training data. In Qadir et al. [70], a framework is created to annotate data that can generate the rest of the segmentation masks with only a few ground truth masks.

#### 2.2.2. Video Artifacts

A problem that still needs to be considered is the influence of video artifacts, such as reflections or blurring, on the detection rate of the methods. Attempts have been made to detect these and use artifact-specific methods to restore the frames; for example, the *Endoscopy artifact detection (EAD 2019) challenge* [71] was also conducted for this purpose.

The article by Soberanis-Mukul et al. [72] examines in detail the impact of artifacts on polyp detection rates. This allowed us to determine the artifact types with the most significant influence. With these, a multi-class model was developed to recognize the different artifact types and the polyps. Since the artifacts were known, regions affected by artifacts could be avoided being wrongly classified as polyps and polyps containing artifacts could be better classified.

#### 2.2.3. Real-Time Application

To support a physician in a real-world scenario, models should be real-time capable, meaning they should achieve a processing speed of about 25 FPS, as colonoscopy videos usually run at 25–30 FPS, and newer systems may run with up to 50–60 FPS. Of course, some speed-up can be achieved by using appropriate hardware. However, concerning real-world use, speed measurement should be performed on hardware reasonable for a physician or hospital.

### 2.3. Data Preprocessing

To ensure high processing speed while maintaining high detection accuracy, we rescale the images to a size of 640 × 640 pixels. This rescaling allows the detection system to be efficient and high performing, maintaining a speed of 20 ms on an NVIDIA RTX 3080 GPU. In the clinical application subsection, we further explain the use of different GPUs and the GPU requirements for a system capable of real-time processing. We empirically tested different image sizes and found the best trade-off between speed and accuracy at a scale of 640 × 640 pixels. Additionally, we transfer the image and model to a half-precision binary floating-point (FP16). Typically, most machine learning models are in precision binary floating-point (FP32). With FP16, the model calculates faster but maintains high-performing results. We found no decrease in performance of the system by decreasing the network to FP16. The next step is image normalization. All image pixels are normalized in the following way: the min–max normalization function linearly scales each feature to the interval between 0 and 1. Rescaling to intervals 0 and 1 is completed by shifting the values of each feature so that the minimum value is 0. Then, a division by the new maximum value is performed (which gives the difference between the original maximum and minimum values).

**Figure 3:**
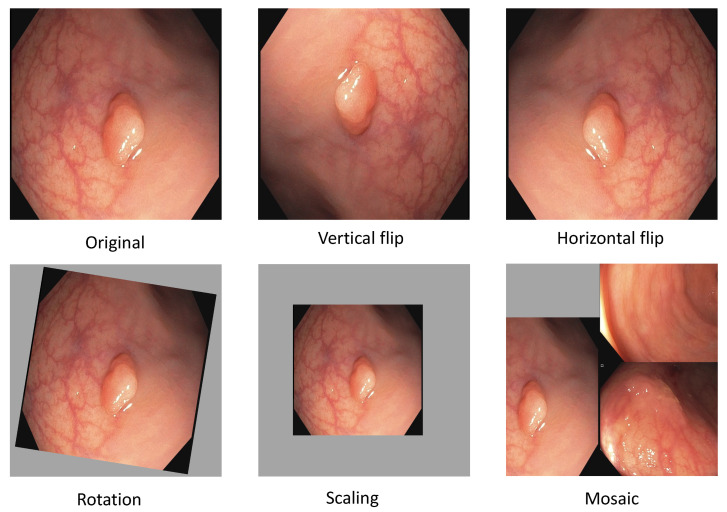
Data augmentation for polyp detection. This figure shows the isolated augmentation we perform to create new training samples. All of these are executed together with a certain probability in our implementation.

The values are transformed element-wise using the following formula:

$$X_{sc}=\frac{X-X_{\min}}{X_{\max}-X_{\min}}$$

where *X* denotes the old pixel value, $X_{sc}$ (X scaled) the new pixel value, $X_{\min}$ the minimum pixel value of the image, $X_{\max}$ the maximum pixel value of the image. After normalization, we apply data augmentation. In deep learning, augmenting image data means modifying the original image data by using various processes. We are applying the augmentation displayed in Figure 3. The most basic augmentation we apply is flip augmentation. This is well suited for polyps as the endoscope can easily rotate during colonoscopy. Here, the image is flipped horizontally, vertically, or both. We applied a probability of 0.3 for up and down flips and a vertical flipping probability of 0.5. We additionally apply rescaling to the image with a probability of 0.638. Rescaling creates polyps in different sizes, adding additional data to our dataset. The translation moves the image along the horizontal axis. Furthermore, we applied a low probability of 0.1 to rotate the image with a randomly created degree. For example, 20-degree rotation clockwise. As the last augmentation, we apply mosaic data augmentation. Mosaic data augmentation mixes up to four images into one image. In this implementation images can not overlap. Thereby, the image is rescaled, causing the images to appear in a different context. Mosaic data augmentation is applied with a probability of 0.944. These data augmentations are only applied to the training data. All of the hyperparameters for these augmentations are chosen by the parameter optimization of the genetic algorithm which is further illustrated in the hyperparameter optimization subsection below. All of the augmentations are combined to create new training images.

### 2.4. Polyp-Detection System

**Figure 4:**
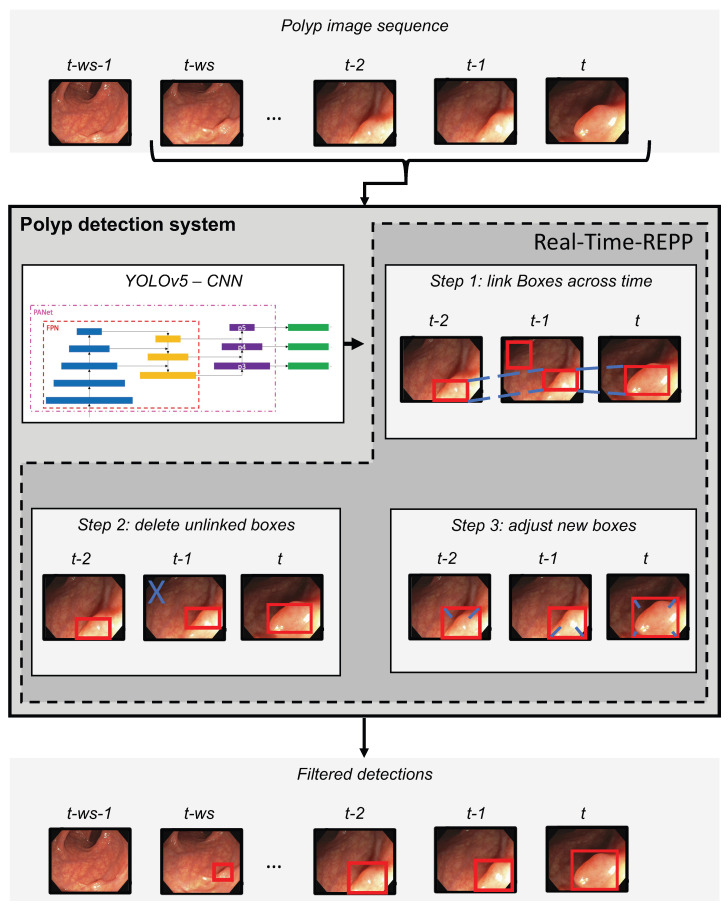
Overview of the polyp-detection system. This figure shows all the steps of the whole polyp-detection system. The start is an input of a polyp sequence ending with the last frame from the endoscope (t). From this sequence, ws frames are extracted and given to CNN architecture. Then detections are performed with YOLOv5, and the predicted boxes are post-processed by RT-REPP. Afterward, final filtered detections are calculated.

As illustrated in Figure 4, the polyp-detection system starts with an input of a polyp image sequence. A polyp image sequence consists of a stream of single images extracted from a grabber of the endoscope in real-time. *t* states the currently processed frame. t−1 denotes the frame before *t*, t−2 the frame before t−1, etc. The parameter ws denotes our new window size, which we introduce to apply real-time robust and efficient post-processing (RT-REPP). The polyp image sequence is now passed to the polyp-detection system. The polyp-detection system consists of two parts: the CNN detection architecture, here YOLOv5, and the post-processing, here real-time REPP (RT-REPP). The trained YOLOv5 model is now predicting boxes and passing those boxes to RT-REPP. RT-REPP consists of three main steps: first, boxes are linked across time steps, i.e., frames. This step is linking boxes according to the linking score. Details on the linking score are displayed in a subsection below. Second, unmatched boxes or boxes which do not meet specific linking and prediction thresholds are discarded through the system. Third, the boxes are adjusted using the predicted boxes from past detections. Finally, the filtered detections are calculated and displayed on the screen.

### 2.5. YOLOv5

**Figure 5:**
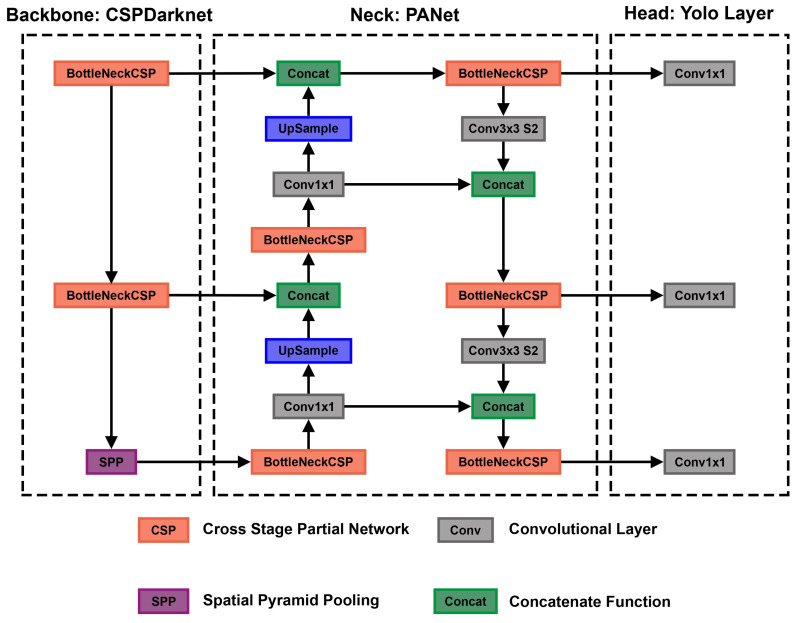
Detailed overview of the YOLOv5 architecture. This overview shows the whole architecture of YOLOv5. The starting point is the backbone CSPDarknet, the main feature extractor (the image is input for the BottleNeckCSP). These extracted features are then given to the PANet neck structure at three stages. Finally, in the head, three outputs are computed. These three outputs are specially designed for small, medium, and large objects and already contain the bounding box predictions. This figure is adapted from Xu et al. [73].

In an end-to-end differentiable network, the YOLOv5 (https://github.com/ultralytics/yolov5, accessed on 18 December 2022) model was the first object detector to connect the technique of predicting bounding boxes with class labels. The YOLOv5 network consists of three main pieces. The neck is a construction of multiple layers that mix and combine image features to pass the prediction forward. The head takes the features from the neck and tries to predict boxes and classes. They use a CNN that aggregates and forms image features at different granularities for the backbone. To create the bounding boxes, YOLOv5 predicts them as deviations from several anchor box dimensions. In Figure 5, we illustrate the YOLOv5 architecture. The objective function of YOLOv5 is defined by minimizing the sum of three losses box-loss, cls-loss, and obj-loss. The box-loss measures how accurately the predicted BBs are drawn around the polyp. The cls-loss measures the correctness of the classification of each predicted bounding box. In our case, it is just one class (polyp). The objectiveness loss (obj-loss) penalizes the model for detecting the wrong bounding box.

#### 2.5.1. CSP as a Backbone

The cross stage partial network (CSPNet) model is based on DenseNet, which was created to connect layers in CNNs to build the backbone for the YOLOv5 model [74]. YOLOv5 created CSPDarknet by incorporating a CSPNet into Darknet. The most significant change of CSPDarknet is that the DenseNet has been reworked to divide the base layer’s feature map by cloning it and sending one copy via the dense block, while sending the other directly to the next stage. Therefore, the CSPDarknet solves the problem of vanishing gradients in large-scale backbones. This is accomplished by splitting the base layer’s feature map into two sections and combining them using a suggested cross-stage hierarchy. The fundamental idea is to separate the gradient flow to propagate over several network pathways. It was demonstrated by varying concatenation and transition phases that the propagated gradient information could have a considerable correlation difference. In addition, CSPNet may significantly minimize the amount of processing required and enhance inference speed and accuracy. CSPDarknet uses two BottleNeckCSPs and one spatial pyramid pooling (SPP) shown in Figure 5. SPP is a pooling layer that removes the network’s fixed size limitation, allowing a CNN to operate with changing input sizes. It aggregates the features and provides fixed-length outputs that are then sent to the next layer or classifier. This works by pooling the results of each spatial bin (like max-pooling). The SSP produces kM-dimensional vectors, with M being the number of bins and k being the number of filters in the last convolutional layer. Therefore, the output is a fixed-dimensional vector. We chose CSP as a backbone for our models using VGG-16 or ResNet50 yields worse results on our validation data than the CSP backbone. Nevertheless, VGG-16 or ResNet50 could also be used as a backbone for this network, as those are valid options for polyp-feature extraction also shown in Tajbakhsh et al. [75] and Sharma et al. [76]. Still, we had the best results using the CSP backbone.

#### 2.5.2. PANet as a Neck

**Figure 6:**
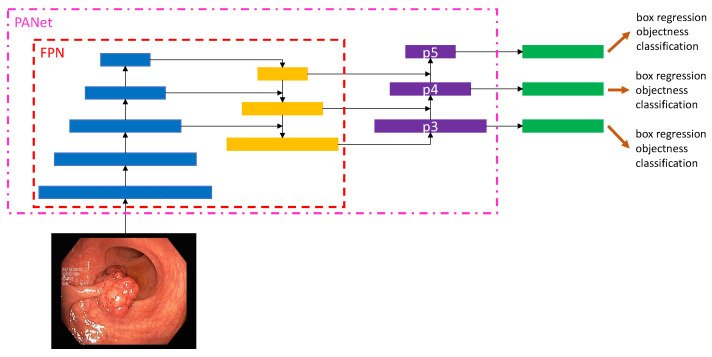
Overview of the PANet of YOLOv5. This overview shows a more detailed view of the PANet structure in YOLOv5. The starting point is a polyp input image. The FPN feature pyramid architecture is illustrated in interaction with the PANet. Finally, three outputs are given. These three outputs are specially designed for small (p5), medium (p4), and large (p3) objects.

The YOLOv5 architecture uses a path aggregation network (PANet) as its neck to improve the information flow [77]. Figure 6 illustrates the PANet and its connections to the architecture in more detail. PANet uses a novel feature pyramid network (FPN) topology with an improved bottom–up approach to improving low-level feature propagation. In the present architecture, the path starts with the output of the SPP from the backbone, which is passed to a CSP. This output is sent into a convolutional layer and is then upsampled. The result is then concatenated with the output from the second CSP in the backbone through a lateral connection and passed through the same combination again, which is then concatenated with the output from the first CSP of the backbone. Simultaneously, adaptive feature pooling is employed to restore broken information paths between each proposal and all feature levels. It is a fundamental component aggregating features from all feature levels for each proposal, preventing outcomes from being allocated randomly. Furthermore, PANet uses fully-connected fusion. These augments mask prediction with small fully-connected layers, which have complementary features to the fully-connected network (FCN) initially utilized by Mask R-CNN, to capture multiple perspectives on each proposal. Information diversity increases and higher-quality masks are generated by combining predictions from these two perspectives. Both object detection and instance segmentation share the first two components, resulting in a much-improved performance for both tasks.

The following steps are used for the adaptive feature pooling. First, the authors map each suggestion to distinct feature levels. Next, a function is utilized to pool feature grids from each level, following Mask R-CNN. The feature grids from different levels are fused using a fusion operation (element-wise max or sum). To integrate features into the network, pooled feature grids are passed through one parameter layer individually in the following sub-networks, followed by the fusion operation. For example, the box branch in FPN contains two fully-connected levels. Between the first and second convolutional layers, the two levels fuse together. For further prediction, such as classification, box regression, and mask prediction, the fused feature grid is utilized as the feature grid of each proposal.

The primary route is a tiny FCN with four convolutional layers in a row and one deconvolutional layer. Each convolutional layer has 256 × 3 × 3 filters, whereas the deconvolutional layer upsamples by two. Like Mask R-CNN, it predicts a binary pixel-wise mask for each class individually to decouple segmentation and classification. A short path from layer conv3 to a fully-connected layer is also created. The network is used with half-precision, cutting the computational cost by halving. A fully-connected layer is employed to forecast a class-agnostic foreground/background mask. It is efficient and allows the fully-connected layer’s parameters to be trained with more data, resulting in improved generality. They employ a mask size of 28 × 28 such that the fully-connected layer generates a 784 × 1 × 1 vector. The mask predicted by the FCN is reshaped to the same spatial size as this vector. The final mask prediction is obtained by combining the masks of each class from the FCN with the foreground/background prediction from YOLOv5. Compressing the concealed spatial feature map into a short feature vector, which loses spatial information, is avoided using just one YOLOv5 layer for final prediction instead of several. Finally, the last YOLOv5 layer creates three different feature maps to provide multi-scale prediction, allowing the model to handle small, medium, and large objects.

##### Hyperparameter Optimization

We use a genetic algorithm to find optimal hyperparameters [78]. The genetic algorithm starts by using YOLOv5 default set of hyperparameters, training the entire YOLOv5 model until 15 epochs, then calculating the F1-score. After that, the hyperparameters are mutated and training is restarted. If the calculated F1-score is higher than the scores in the past, the best score and its hyperparameters are saved. After iterating over 10,000 genetic optimizations, we found our final parameters and retrained the algorithm for 54 epochs with the corresponding hyperparameters. At this point, the algorithm is stopped through early stopping.

##### Model Selection

We tested different architectures to select the best polyp detection model. We used a 5-fold cross-validation on our training data to determine the final model. We used 80% of the data for training and 20% of the data for validation. The cross-validation results are shown in Table 3. The best results are achieved in precision, recall, F1, and mAP using the YOLOv5 model. Still, the model keeps real-time capability while having an average number of parameters compared to the other models.

### 2.6. Robust and Efficient Post-Processing (REPP) and Real-Time REPP (RT-REPP)

**Figure 7:**
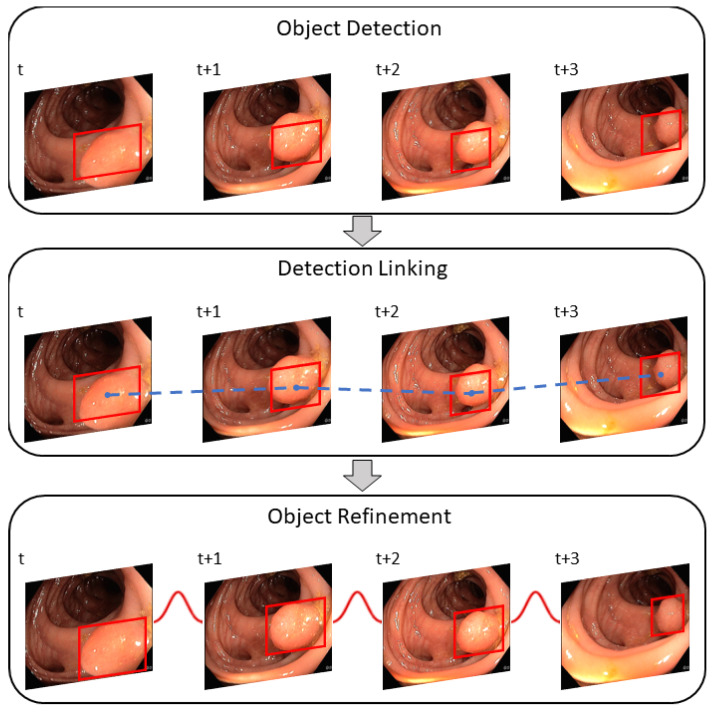
The REPP modules used for video object detection post-processing. The object detector predicts a polyp for a sequence of frames and links all bounding boxes across frames with the help of the defined similarity. Lastly, detections are refined to minimize FPs. This figure is adapted from Sabater et al. [13].

Image object detectors process each frame of a video individually. Each frame of an incoming stream of frames is viewed independently of the previous and subsequent frames. As a result, information is lost and the performance of such detectors can significantly differ between images and videos. Moreover, video data confronts the object detector with unique challenges, such as blur, occlusion, or rare object poses. To improve the results of the object detector for video data, the post-processing method REPP [13] relates detections to other detections among consecutive frames. Thus, the temporal dimension of a video is included. REPP links detections across consecutive frames by evaluating their similarities and refining their classification and location. This helps to suppress and minimize FP detections. The algorithm can be divided into three modules: (1) object detection, (2) detection linking, and (3) detection refinement. Figure 7 shows an overview of the REPP modules.

#### 2.6.1. Object Detection

Object detection works on any object detector that provides bounding boxes and a class confidence score. For each frame *t*, the detector delivers a set of object detections. Each detection $o_{t}^{i}$ is described by a bounding box (bb), semantic information and the appearance of the patch (small piece of an image). The bounding box is defined as $bb_{t}^{i}=\left\{x,y,w,h\right\}$, where *x* and *y* is the upper left corner, *w* the width, and *h* the height of the bounding box. Semantic information, such as the vector of class confidences, is defined as $cc_{t}^{i}\in R^{C}$ with *C* for the number of classes and a L2-normalized embedding $app_{t}^{i}\in R^{256}$ which represents the appearance of a patch.

#### 2.6.2. Detections Linking

Linking detections along the video are created by a set of tubelets and continue as long as corresponding objects are found in the following frames. A similarity function is used to link two detections between two consecutive frames.

(1) $$\begin{array}{cc} f_{\mathrm{loc}} & =\{\mathrm{IoU},d_{\mathrm{centers}}\} \end{array}$$

(2) $$\begin{array}{cc} f_{\mathrm{geo}} & =\{{\mathrm{ratio}}_{w},{\mathrm{ratio}}_{h}\} \end{array}$$

(3) $$\begin{array}{cc} f_{\mathrm{app}} & =d_{\mathrm{app}} \end{array}$$

(4) $$\begin{array}{cc} f_{\mathrm{sem}} & =f_{\mathrm{sem}}^{a}\cdot f_{\mathrm{sem}}^{b} \end{array}$$

where $f_{\mathrm{loc}}$ is the location which is specified through the Intersection over Union (IoU) and the relative euclidean distance between two bounding box center points ($d_{\mathrm{center}}$). The IoU indicates the overlap between two boxes. The larger the overlap, the more likely it is that both boxes mark the same object.

In addition, the distance between the two center points is used. $f_{\mathrm{geo}}$ is the geometry of the bounding boxes, which is defined as the ratio of width (${\mathrm{ratio}}_{w}$) and height (${\mathrm{ratio}}_{h}$) between the two bounding boxes. This score is high if both boxes have a similar size. $f_{\mathrm{app}}$ is the appearance similarity in which the Euclidean distance between the appearance embeddings ($d_{\mathrm{app}}$) are calculated. A more similar appearance results in a higher score. Lastly, $f_{\mathrm{sem}}$ is the dot product of the class confidence vectors $cc_{t}^{i}$. The greater the confidence vectors of both detections, the more likely it is that both boxes mark a polyp. Using these features, a link score (LS) is calculated between two detections.

(5) $$LS\left(o_{t}^{i},o_{t+1}^{j}\right)=f_{\mathrm{sem}}X\left(f_{\mathrm{loc}},f_{\mathrm{geo}},f_{\mathrm{app}}\right)$$

Thereby, X is a function for logistic regression trained so that it can differentiate if two detections belong to the same object instance. In the following, the linking process is algorithmically explained.

Algorithm 1 shows a general description to obtain pairs of frames. The algorithm uses a list of predictions, processes each frame, calculates the distance between objects in both frames, and saves the value in a distance matrix. The objects with the lowest distance are then considered a pair, and a list of pairs is returned.
**Algorithm 1** Get a list of pairs of frames that are linked across frames1:**function**getPairs(predictions)2:    **for** index← 0 **to** count of frames **do**3:        predsFrame1 ← predictions[index] ▹ Get frame predictions from current index4:        predsFrame2 ← predictions[index+1]   ▹ Predictions of next frame5:        framePairs ← empty list6:        **if** length(predsFrame1) ≠0 **and** length(predsFrame2) ≠0 **then**7:           distances ← 2D-Array with 0 for each cell8:           **for** i←0 **to** length(predsFrame1) **do**9:               **for** j←0 **to** length(predsFrame2) **do**10:                   distances[*i*][*j*] ←LogReg(predsFrame1[*i*], predsFrame2[*j*])11:               **end for**12:           **end for**13:           framePairs ← solveDistances(distances)14:        **end if**15:        pairs.append(framePairs)16:    **end for**17:    **return** pairs18:**end function**

Next, tubelets are created (Algorithm 2) from a list of linked pairs. Tubelets link all bounding boxes that identify the same object across a series of frames.
**Algorithm 2** Tubelets creation from list of linked pairs1:**function**getTubelets(predictions, pairs)2:    tubelet ← empty list3:    **for each** frame **do**4:        **for each** pair in following frames **do**5:           **if** frame has no pair **then**6:               start new tubelet7:           **end if**8:           **if** frame has pairs **then**9:               append box from pair to tubelet10:           **end if**11:        **end for**12:    **end for**13:    **return** tubelets14:**end function**

#### 2.6.3. Object Refinement

The use of tubelets improves the classification and location. The first step of tubelet creation is recalculating the detection classification scores. Therefore, all class confidence vectors are averaged and assigned to each detection within the tubelet (see Algorithm 3). This helps correct mislabeled detections and disambiguate those with low confidence.
**Algorithm 3** Rescore tubelets1:**function**rescoreTubelets(tubelets)2:    **for each** t∈ tubelets **do**3:        $s_{avg}=\frac{1}{\left|t\right|}\cdot \sum_{p\in t}s_{p}$   ▹ Average score *s* of predictions *p* of tubelets4:        $\forall p\in t:s_{p}=s_{avg}$     ▹ Assign average to all prediction scores5:    **end for**6:    **return** tubelets7:**end function**

The next step is to improve the detection positions. Each coordinate of a linked object is treated as a noisy time series. Smoothing is used to alleviate the noise with the help of a one-dimensional Gaussian filter convolution along with each time series. The smoothed series are then used as the set of coordinates of the object in the tubelet (Algorithm 4, line 7).
**Algorithm 4** REPP1:**function**REPP(objectDetectionPredictions)   ▹ Gets all predictions from detection network2:    videoPredictions ← filterPredictions(objectDetectionPredictions)3:    pairs ← getPairs(videoPredictions)4:    tubelets ← getTubelets(videoPredictions, pairs)5:    tubelets ← rescoreTubelets(tubelets)6:    **if** recoordinate == True **then**        ▹ Tubelets reccordination is optional7:        tubelets ← recoordinateTubelets(tubelets)8:    **end if**9:    predictions ← tubeletsToPredictions(tubelets) ▹ Convert to specific format10:    **return** predictions11:**end function**

The final REPP algorithm (Algorithm 4) is a combination of all aforementioned algorithms executed in order. First, filter detection predictions (line 2), then obtain all pairs (line 3) and afterward compute the tublets out of the pairs (line 4). Then rescore detections within tubelets (line 5) and recoordinate them for improved prediction results (line 7). Lastly, filter all predictions that do not reach a certain threshold and convert them to a specific prediction result format, such as the COCO format (line 9).

Since REPP is a post-processing method that only works on finished videos, REPP includes past and future frames. We modified the code for real-time application to only include past frames, calling the algorithm RT-REPP. To compare REPP and RT-REPP in our real-time application, we included a buffer of pre-defined length to run the original REPP. The size of the buffer is adjustable to fit the available resources. The greater the length, the longer it takes to execute REPP. Before REPP is executed, the buffer must be completed, which causes a size-dependent delay at the beginning of each video. To overcome this delay, REPP is run from the start frame and executed for every new frame until the buffer is completed. The completed buffer is passed, and the oldest frame is deleted as a new frame is inserted. Since the delay times for our application are relatively short, we accept this short delay. See Algorithm 5 to understand the basic workflow of RT-REPP. We define a window size ws, which determines the window length. A buffer size ws of 300 is sufficient for a real-time stream of 25 FPS. A ws of more than 300 does not improve the accuracy significantly.
**Algorithm 5** RT-REPP1:**function**RT-REPP(framePrediction)2:    **if** buffer is full **then**3:        delete oldest frame4:    **end if**5:    add prediction to buffer6:    run REPP on buffer7:**end function**

**Figure 8:**
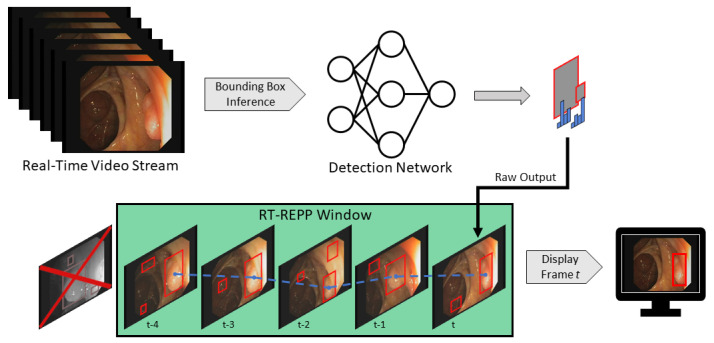
Real-time REPP. It obtains a stream of video frames, where each frame is forwarded into a detection network. The result of the current frame is stored into the buffer (green) and REPP is executed afterward. The improved result are then displayed.

To implement RT-REPP in our real-time system, we combined C++ and python. We used the lightweight header-only library pybind11, which allows us to use C++ types and methods in python and vice versa. To our knowledge, REPP and RT-REPP have not been used before in the domain of polyp detection. In Figure 8, the workflow of real-time REPP is illustrated.

We tested different linking-score thresholds and window sizes to choose the hyperparameters of RT-REPP. The boxes scoring below the linking-score threshold are removed from the final detection results. As described early, we set the window size to 300. We tested different linking-score thresholds. Our results determined a score of 0.2 to be the most effective.

### 2.7. Clinical Application

To develop a system for clinical trials, it is mandatory to understand the current settings of examination rooms. Endoscopic and other medical equipment are complex devices with intricate setups. Therefore, this is only a brief overview of these highly sophisticated components.

**Figure 9:**
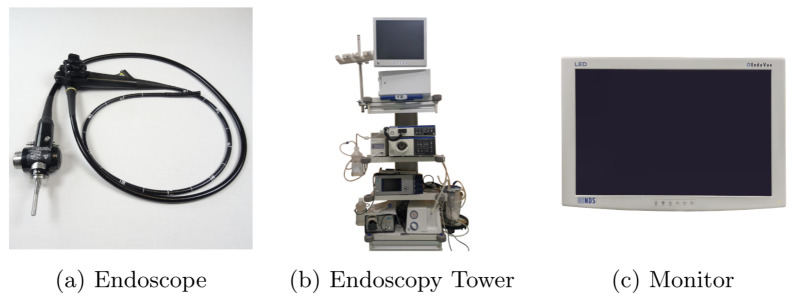
This figure illustrates the setting for the examination room.

Figure 9 shows an example of medical devices used during endoscopic interventions. Figure 9a presents an endoscope composed of a flexible tube, controlled and operated by physicians during examination through several control buttons and physical force. A fish-eye camera is on the tip of this tube, combined with a light source to capture an RGB video stream. The endoscopy tower contains the entire endoscopic equipment and, most importantly, the camera’s light source and an endoscopy processor (Figure 9b). The endoscopic processor captures the camera stream and processes it into a regular video signal. This signal can be displayed on a monitor, as shown in Figure 9c. These components provide physicians with real-time visual feedback during endoscopic interventions. Based on the given setting, we developed a prototype for clinical application. Instead of directly connecting the endoscopy processor to the monitor, our system stands between the processor and monitor, processing all frames before forwarding them to the monitor.

Table 4 shows the main hardware components of our system, which allows for real-time image processing. However, these are just as important as a suitable software. In order to provide physicians with the best possible user experience, all incoming frames must be displayed as fast as possible to minimize latency. To this end, image capturing, displaying, and processing are running in separate threads. The first thread uses the Blackmagic SDK to capture frames, which depends on the frame rate. For instance, Olympus CV-190 provides 50 FPS, receiving a frame every 20 ms. Therefore, it is essential to distribute the additional workload on other threads. If only one thread is used, incoming frames are buffered, resulting in an overall delay across all related threads. Considering this, thread one only captures and transforms incoming data to an OpenCV matrix, passing it to subscribing pipelines.

**Figure 10:**
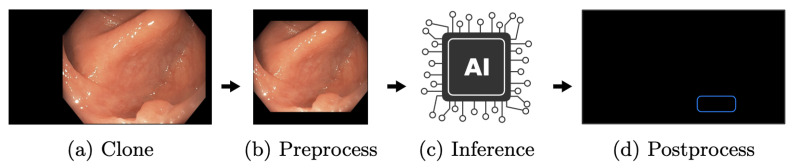
The AI pipeline. This figure depicts the AI pipeline used to apply the created polyp-detection system in a clinical environment.

One receiver is the AI pipeline, shown in Figure 10. In this thread, all incoming frames are cloned (Figure 10a) to ensure that all operations on those image matrices do not interfere with other processes. The clone shown in (Figure 10b) is preprocessed. Here, the frame matrices are transformed to fit the AI network. First of all, the black borders of the images are cropped. In Figure 10a to Figure 10b this is illustrated. The resulting 640 × 640 matrix is transformed from BGR to RGB and uploaded to GPU memory. Here, the matrix is processed through YOLOv5 (Figure 10c). Based on the input, it results in relative coordinates, classes, and scores for every detection. The last step is a transformation resulting in a vector of quadruples, containing xy-coordinates, width, and height of bounding boxes to suit the original matrix (Figure 10d). Under consideration of thresholds, detections with low confidence are removed, while the remaining detections are transformed and forwarded to the display pipeline.

**Figure 11:**
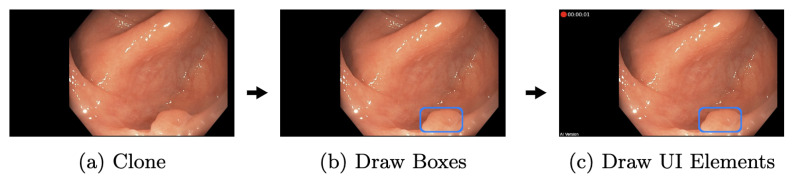
The display pipeline. This figure depicts the display pipeline used to display the final detection results to the gastroenterologist.

The independent display pipeline thread is designed to display captured frame matrices as fast as possible. Like the AI pipeline, matrices are cloned at the beginning, shown in Figure 11. Consequently, no processing is applied on the original matrices; therefore, other pipelines remain unaffected. Then, based on the most recent detections of the AI, new boxes are drawn and old ones removed. The boxes remain on the screen until a new cycle of the AI pipeline has finished. Additionally, a few extra UI elements, such as a timer, indicating that the AI is running before frames are forwarded and displayed. This design, as mentioned earlier, decouples the AI and display pipeline. Hence, a slower AI does not directly result in higher display latency. Nevertheless, the performance of the AI pipeline remains an essential factor. Faster executions lead to more inferences and, therefore, more precise boxes, given that the displayed frame is closer to the AI pipeline frame.

The prototype was tested on two different GPUs to show the performance differences during clinical application. The prototype components are listed in Table 4. A second computer streamed a colonoscopy video instead of an endoscopy processor, just like an endoscopy processor does. Meanwhile, the prototype captured the signal, as mentioned earlier. This ensures identical, reproducible conditions and guarantees occurring polyps during the experiment. The prototype is not able to distinguish this method from a live endoscopy. The streamed examination video is presented in 1920 pixels and 50 fps, equivalent to streams of Olympus CV-190. Our test used the MSI GeForce RTX 3080 Ti, an up-to-date high-end GPU released on 3 June 2021. The NVIDIA Geforce GTX 1050 Ti, a low-budget GPU two generations ago, was used for a second test run. This GPU was released on 27 May 2016. All other hardware components and software parts were constant throughout testing.

In the setting of Table 5 5000 frames are considered. Out of those 5000 frames, the RTX 3080 Ti executed the AI pipeline 2996 times. At the same time, the GTX 1050 Ti made 313 executions. This is based on the AI’s average execution time (AI pipeline average execution time) 19.5 ms and 306.7 ms, respectively. During the usage of RTX 3080 Ti, there was a 15-fold performance gain. The AI pipeline was applied on every 1.7th frame on this GPU, while only every 16th frame was evaluated through the inferior GPU. Considering those results and a synchronized display pipeline, it takes two frames until bounding boxes are displayed. Furthermore, those two boxes remain displayed for two more frames until they are updated again, resulting in a total display time of four frames (80 ms) for the RTX 3080 Ti. In comparison, the GTX 1050 Ti accumulates 32 frames (640 ms), while 16 frames (320 ms) are needed to generate the first bounding box. This does not illustrate the worst or the best-case scenario.

**Figure 12:**
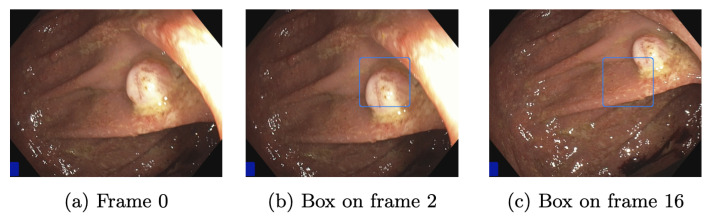
Detection shift through latency.

An example was created to show the delay in the appearance of a bounding box. Figure 12a shows a frame forwarded to the AI pipeline. Since the RTX 3080 Ti needs an average of 1.7 frames, bounding boxes appear in frame two. This is illustrated in Figure 12b. While the camera moves, frame two is slightly shifted to the bottom, but the polyp is still mainly boxed. The GTX 1050 Ti takes an average of 16 frames, shown in Figure 12c. The polyp is mainly outside the bounding box. A box might appear based on the speed at which the endoscope is moved, even if a polyp is no longer displayed. This is highly unlikely for the RTX 3080 Ti, which in the best case, shows a bounding box on the consecutive frame. This delay must be considered when using slower GPUs but can be neglected if the endoscope’s withdrawal motion is made slowly.

The test shown in Table 5 has been performed on an actual prototype. Therefore, the software has not been altered. In addition, a video was recorded simultaneously, and this is done for quality assurance and to retrieve additional test data. The recording pipeline is independent, but the GPU is used for H.264 video encoding, which causes an additional load and can affect the performance of the AI pipeline. In general, our prototype is not designed for a specific GPU, all NVIDIA GPUs with CUDA compatibility over the last five years can be used, but it will affect the user experience. In an actual examination, prototypes have been used with a MSI GeForce RTX 2080 SUPER Ventus XS OC with no significant change in user experience.

## 3. Results

For the evaluation, we use two datasets. The CVC-VideoClinicDB dataset is the first benchmark dataset for polyp detection in videos [59]. The previous benchmark datasets, e.g., ETIS-Larib and CVC-ColonDB, only allow a comparison based on still images. The CVC-VideoClinicDB dataset has the ability to evaluate models on video data, which is a more realistic scenario, as real polyp detection outputs from endoscopies are not images but a stream of images provided in real-time. As our architecture explained in methods only applies to videos or a stream of images, we chose the CVC-VideoClinicDB dataset as our main evaluation dataset. The second dataset is our own test dataset called EndoData. In the CVC-VideoClinicDB dataset, the polyp sequence begins with the polyp already in view in the first frame. Since our dataset contains the entire footage, the polyps appear further into the image sequence. Therefore, the EndoData dataset emulates the clinical practice more closely, which makes the evaluation even more realistic in the application. We can additionally calculate a metric measuring the time taken to detect a polyp. We published the code for the application and evaluation of our system on our webpage (https://fex.ukw.de/public/download-shares/d8NVHA2noCiv7hXffGPDEaRfjG4vf0Tg, accessed on 18 December 2022).

The F1-score evaluates the model’s quality. The F1-score describes the harmonic mean of precision and recall as shown in following equations:

$$\mathrm{Precision}=\frac{TP}{TP+FP}\;\;\;\;\mathrm{Recall}=\frac{TP}{TP+FN}$$

$$F_{1}=\frac{2*\mathrm{Precision}*\mathrm{Recall}}{\mathrm{Precision}+\mathrm{Recall}}=\frac{2*TP}{2*TP+FP+FN}$$

We count an annotation as TP if the boxes of our prediction and the boxes from the CVC-VideoClinicDB dataset ground truth overlap at least 50%. Additionally, we choose the mAP, which is a standard metric in object detection [80]. The mAP is calculated by the integral of the area under the precision-recall curve. All predicted boxes are first ranked by their confidence value given by the polyp-detection system. Then we compute precision and recall for different thresholds of these confidence values. When reducing the confidence threshold, recall increases and precision decreases. This results in a precision–recall curve. Finally, the area under the precision–recall curve is measured. This measurement is called the mAP. Furthermore, our approach introduces new parameters to the polyp-detection system. One of the parameters is the width of the detection window ws.

We created the following evaluation on our dataset (EndoData). Our evaluation considers two baseline models: the YOLOv5 (base) model and the Faster R-CNN baseline. The YOLOv5 (base) model is the basic YOLOv5 model trained on the EndoData dataset without any hyperparameter optimization, data augmentation, post-processing, or other changes for polyp detection. The second baseline model is a Faster R-CNN with a ResNet-101 backbone. This involves training a Faster RCNN with default parameters using the Detectron2 framework [81].

Furthermore, we show three different stages of our polyp-detection system. First, YOLOv5 advanced (adv.), which is training the YOLOv5 model but with all our in section methods explained features and optimization to specialize it for the polyp detection task. Second, REPP is a trained YOLOv5 (adv.) model, including the REPP post-processing. This is not applicable in real-time, as the REPP algorithm only works on recorded videos. Afterward, we introduce the RT-REPP. The RT-REPP is our version of REPP, which works in real-time. Our polyp-detection system ENDOMIND-Advanced is in the following evaluation, referred to as RT-REPP. All models are trained on our training data using four Quadro RTX 8000 NVIDIA graphics cards, and the test application is made on an NVIDIA GeForce RTX 3080. The results of these models are shown in detail in Table 6, Table 7, Table 8, Table 9, Table 10, Table 11 and Table 12.

### 3.1. CVC-VideoClinicDB Data Evaluation

To compare our polyp-detection system to the published research, we use the publicly available CVC-VideoClinicDB dataset. To our knowledge, the best-performing algorithm on the dataset was published by Qadir et al. [55]. Therefore, Qadir et al. [55] is included in the evaluation in Table 6. In Table 6, different baseline and advanced stage models are compared. All values are calculated according to the CVC-VideoClinicDB challenge norm. The CVC-VideoClinicDB challenge norm defines the same calculations used for calculation in the GIANA challenge 2017 [60]. Therefore, the means are calculated by summing all the results for every image and dividing the sum by all images in the dataset (micro mean). We use this micro mean structure throughout this paper. All presented means are micro averages over all images. We excluded video 18 from the CVC-VideoClinicDB dataset, because 77 of 381 images are labeled incorrectly.

For the F1-score, REPP has the highest F1 score. However, REPP is not applicable in real-time as it is calculated by combining past, present, and future predicted boxes. Therefore, REPP can only be used on recorded videos. We like to include it in the comparison to show the enhancements using the full algorithm. RT-REPP achieves the second-best F1-score and functions in real-time. Using RT-REPP vs. YOLOv5 (adv.) improves the results by a F1-score of 4.15%. The baseline models Faster R-CNN and YOLOv5 (base) achieve lower F1-scores.

Overall our results show that using our hyperparameter, data augmentation, and training setup increases the F1 and mAP by 6.05% and 4.78%. By leveraging our implementation, RT-REPP results improve further by 4.15% and 3.14%. REPP and RT-REPP cause a minimal speed reduction, resulting in roughly a 1 FPS speed reduction for RT-REPP and a 2 FPS reduction in REPP. Therefore, those algorithms can easily be added to neural networks without losing much processing time.

For the detailed evaluation, we computed the mAP and F1-score for each of the 17 videos of the CVC-VideoClinicDB dataset. REPP-RT detects most videos with a F1-score of over 90%. Only videos 3, 5, 7, 12, 15, 17 have a score lower than 90%. These videos also have inferior test results. Negative examples are video 12, with a score of 57.14%; video 17, with a score of 65.72%; and video 15, with a score of 71.79%. We analyze those videos in more detail in the discussion section. The YOLOv5 baseline model also has inferior results with a value of 46.22% and a detection value lower than 50%. Comparing our approach to Jha et al. [48], we achieve better results on the CVC-VideoClinicDB data. However, the Jha et al., model is also capable of polyp segmentation and the system’s speed is faster (180 FPS).

### 3.2. EndoData Evaluation

**Figure 13:**
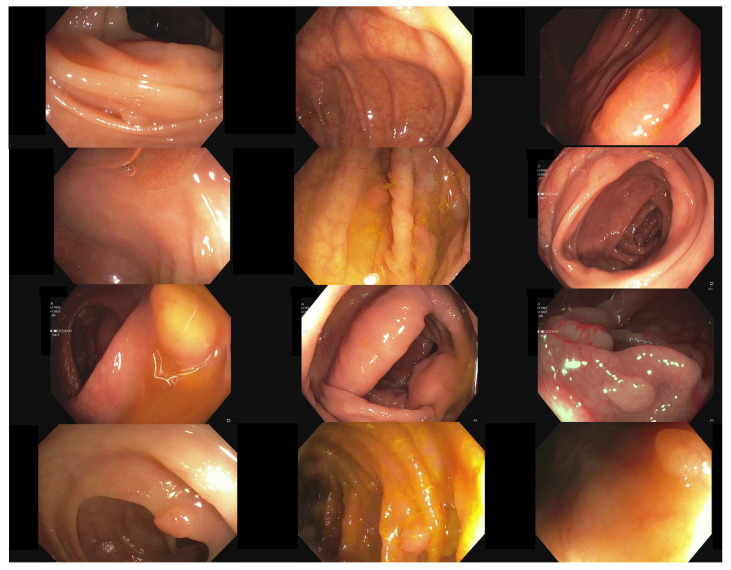
Example images of the Endodata dataset for evaluation.

Our own validation set (EndoData) allows us to detect polyps more precisely and accurately. Table 8 shows an overview of the videos in the dataset and Figure 13 shows examples of the dataset. The EndoData dataset records sequences as the polyp appears in the scene. Therefore, polyps are marked precisely with their first appearance. In comparison, the polyp sequence of the CVC-VideoClinicDB dataset might not start when the polyp is already detected. Those early seconds are crucial as the gastroenterologist has to identify and not miss the polyp during this time. If the polyp is not detected in the early sequence, it increases the risk of missing it. As we like to focus on this early detection, we introduce a second metric that can just be evaluated with a dataset like ours. This metric marks the seconds from first seeing the polyp to first detecting the polyp. We call it first detection time (FDT). Additionally, we compute the FPs and the false positive rate (FPR) per video (Table 10 and Table 11).

The evaluation for FDT is shown in Table 10. For the YOLOv5 (base), only video 4 does not receive a delay in detection. Nevertheless, all polyps are detected at least once with every algorithm. The FDT of YOLOv5 (base) is inferior in all videos to the other models. The Faster R-CNN algorithm does recognize the polyp in the first frame in videos 1, 3, 4, and 10 for YOLOv5 (adv.), REPP, and RT-REPP. The FDT for these three models does not differ except for video 7. This difference is due to REPP and RT-REPP removing the detection in the post-processing process. Those three approaches also detect the polyps in the first frame for videos 1, 3, 4, and 10, like Faster R-CNN. For 9 out of the 10 videos, FDT is under 1 s; therefore, the polyp should be sufficiently detected to show the gastroenterologist its position. Nevertheless, in video 7 there is a FDT of 2.6 s. Such a late detection of a polyp may miss the polyp for the gastroenterologist. However, REPP and RT-REPP are reducing the number of FPs from an average of 113.5 to 78.3 and 91.3.

We evaluate the models on our dataset with the same metrics as the CVC-VideoClinicDB dataset. On the EndoData dataset, the results are equivalent to the predictions of the CVC-VideoClinicDB data. The mAP is, on average, consistently lower than the F1-score. Additionally, REPP is again the best scoring model. Again most values are over 90% F1 value for RT-REPP. The dataset appears to be more challenging than the CVC-VideoClinicDB dataset as there are just five videos with F1-scores over 90%.

Furthermore, we like to compare our results to the results of Livovsky et al. [50]. Livovsky et al., have only evaluated their approach on a closed custom dataset; therefore, we are unable to provide a qualitative comparison on the CVC-VideoClinicDB benchmark. Nevertheless, we qualitatively compare their results with our results on EndoData. Livovsky et al., achieved a recall of 88.5 % with polyps visible longer than 5 s on their custom test data. Our approach achieves a recall of 89.32 % on our custom dataset. As the two test datasets are different it is not possible to quantitatively show which system is better, nevertheless, both systems achieve similar recall values on their test sets.

### 3.3. Explaining the Model with Heatmaps

This paragraph presents a methodology to generate visual explanations for deriving insight into our polyp-detection systems decisions using the Grad-CAM algorithm [82]. We follow the Checklist for Artificial Intelligence in Medical Imaging (CLAIM) [83]. Nevertheless, we changed the Grad-CAM algorithm to fit an object/polyp detection task instead of classification. The Grad-CAM algorithm receives the image of the model’s prediction, the result, and the last two layers of the CNN YOLOv5. YOLOv5 outputs the detections for the three scales p3 (large), p4 (medium), and p5 (small). Each detection output with a shape of $\left[bsz;n_{a};h;w;\left(5+n_{c}\right)\right]$, where bsz is the batch size, $n_{a}$ is the number of anchor boxes per grid cell, *h* is the height of the feature map, *w* is the width of the feature map, four box coordinates + objectness = 5, and $n_{c}$ is the number of classes. Next, the three scales are concatenated and reshaped, resulting in an output of shape $\left[bsz;n_{a}\times h\times w;\left(5+n_{c}\right)\right]$ followed by data augmentation. The augmentation identifies the scales from which the detections originate.

After that, the methodology employs a customized version of the non-max suppression algorithm (NMS) to reduce the number of detections to the most probable ones. For this purpose, the algorithm multiplies objectness probability $p_{o}$ and class probability vector $p_{c}$ and takes its maximum, $p_{d}^{*}=\max\left(p_{d}\right)=\max\left(p_{o}*p_{c}\right)$, which it subsequently uses as one decision criterion for reducing detections. This procedure ultimately results in significantly fewer and more confident detections. Furthermore, it associates each detection with a unique detection probability $p_{d}^{*}$, objectness probability $p_{o}$, and class probability $p_{c}^{\left(i\right)},i=1\cdots n_{c}$. The presented methodology carries these values along and inserts them in the Grad-CAM algorithm for $y^{c}$.

The next step encompasses the execution of the Grad-CAM algorithm for each of the probabilities mentioned above. Here, the proposed methodology calculates for each probability the gradients $\frac{\partial y^{c}}{\partial A^{k}}$ for three feature map activations, namely for p3, p4, and p5.

Afterward, the presented approach transforms the emitted localization maps into heatmaps, which are significantly smaller than the original size of the input image. The proposed method upscales to the original image size by interpolation and then superimposes the heatmaps onto the original image. The resulting image shows highlighted image regions that contain pixels that positively influence the value of $y^{c}$. The method also draws a corresponding bounding box for each detection, its score, and the originating scale onto the superimposed image to increase the informational content. The final result is |#scores|×|#dets|×|#scales| superimposed images for each input image.

YOLOv5 was implemented in the python programming language with the PyTorch deep learning library. For this reason, this work also uses python and PyTorch to implement the Grad-CAM algorithm for YOLOv5 and necessary extensions. The most important feature of the PyTorch deep learning library is the concept of so-called hooks, which enable the extraction of the gradients obtained via backpropagation. Itoh et al. [84] showed that the classic Grad-CAM application may result in noisy heatmaps when using a YOLOv3 algorithm. We achieved less noisy heatmaps by recreating the Grad-CAM algorithm as described above. These heatmaps are similar to the results of Itoh et al., when using their application [84].

**Figure 14:**
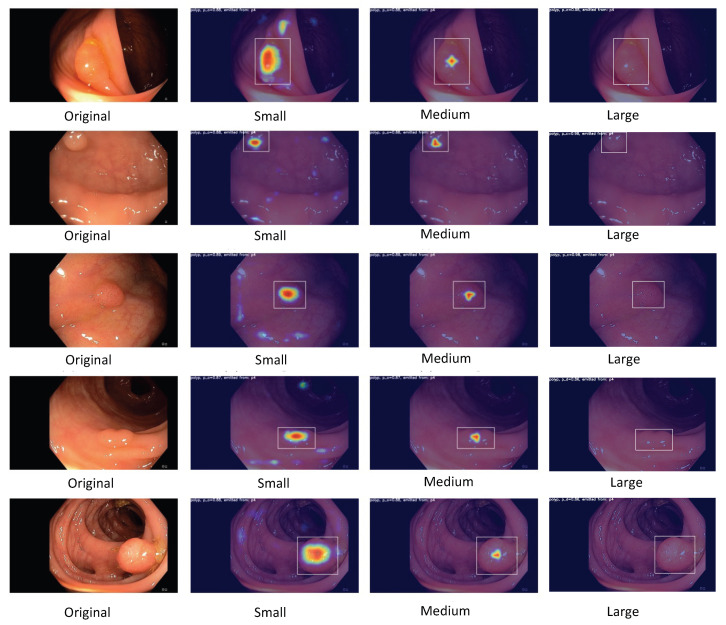
Heatmaps for polyp detection. This figure illustrates the detections of the model using the Grad-CAM algorithm. Thereby, the pixels most relevant for the detection are marked in warm colors such as red, and pixels less relevant for the detection in cold colors such as blue. The CNN has three detection outputs for small, medium, and large objects.

The first column of Figure 14 shows the five original frames on which the model should detect a single polyp. The second, third, and fourth columns in Figure 14 depict the resulting heatmaps of the approach when assigning $p_{c}$ to $y^{c}$ for backpropagation of the respective gradients.

Figure 14 with a small and 14 medium polyp depicts the following behavior: the model focuses on the crucial image regions to classify and localize the present polyp while traversing from the output scale from small to medium. As expected, the model increases the pixel intensity important for the localization from p3 to p4. Furthermore, we notice that the highlighted red regions in Figure 14 encompass the center point of the respective bounding box. This shows that the model’s focus in case of high polyp-detection confidence activates the necessary areas of the image.

Nevertheless, Figure 14 for large polyps displays the opposite behavior where the detected polyps are not highlighted in the heatmaps. The detected polyps are not large enough to activate the neurons for this part of the YOLOv5 architecture.

The above observations conclude that the model is functioning as expected. This shows the necessity of the proposed method to confirm or rebuke the assumption of the analyzed model’s function and expected behavior.

## 4. Discussion

The system’s limitations and clinical use are discussed in the following subsections. We especially focus on false polyp detections and discuss those system failures using frames of our CVC-VideoClinicDB and EndoData datasets. Additionally, we debate the clinical application of the system.

### 4.1. Limitations

**Figure 15:**
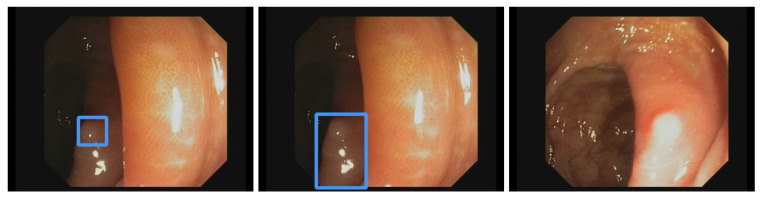
Examples of errors in video 12 of the CVC-VideoClinicDB dataset. The left image shows a correct polyp detection, the middle image misidentifies the size of the polyp and the right image shows no detection due to oversaturation.

We initiate the discussion of our limitations with a failure analysis of our model. First, we refer to Table 7 and Table 12, and specifically to videos with significantly worse performance than the rest, i.e., videos 8, 12, 15, and 17 of the CVC-VideoClinicDB dataset and video 7 of EndoData. The videos differ in polyp detection difficulty; some videos only contain typical polyps with a straight angle and good lighting, while other videos have bad contrast, slanted angles and atypical polyps. Hence, multiple reasons can be attributed to the decreased performance on these videos:

Contrast and lighting are one of the main causes for missing or misidentifying a polyp. Figure 15 shows three frames taken from video 12 of the CVC-VideoClinicDB dataset. The image on the left shows a correct polyp detection and represents the exception. Most other frames either misidentify the size of the polyp, as seen in the middle image or do not detect the polyp at all, as seen in the right image. In this case, it is most likely an issue of contrast, as the polyp is oversaturated. As this applies to most video frames, the F1-score is negatively impacted.

**Figure 16:**
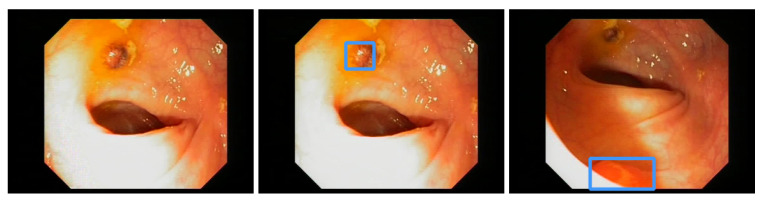
Examples of errors in video 15 of the CVC-VideoClinicDB dataset. The left image shows a missed polyp and the middle image a proper detection. On the right image, another polyp in the same frame is detected, while the other is missed.

Some polyps have uncommon shapes, an atypical surface texture, or a rare color and are underrepresented in the dataset. A notable example of such a polyp can be seen in video 15 of the CVC-VideoClinicDB dataset with some frames shown in Figure 16. Due to its peculiar appearance, this polyp is missed in most frames, especially in those frames containing another polyp, as seen in the right image. However, this is partly caused by the CVC-VideoClinicDB ground truth. The ground truth masks cover only one polyp at a time, even if both are visible in a frame. Rare polyps are a challenge for every supervised model, and this issue can only be improved by collecting more data.

**Figure 17:**
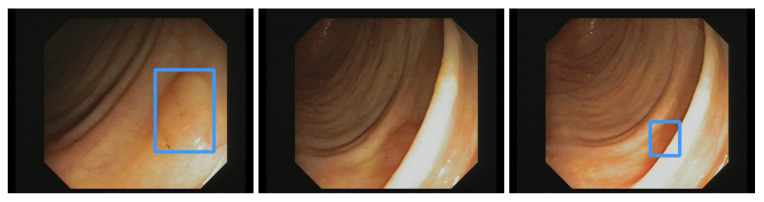
Examples of errors in video 17 of the CVC-VideoClinicDB dataset. The left image shows the detection of a flat polyp. The middle image shows the same polyp being missed because it is blocked by the colon wall. The right image shows a (short) re-detection.

As mentioned above, even typical polyps can be difficult to detect when obstructed by parts of the colon or due to bad lighting and angles. Figure 17 depicts these issues. Furthermore, small polyp size and color that is very similar to the surrounding colon lead to a general miss-rate of around 10% of the polyps frames.

FPs account for a large number of errors and, in turn, diminish the model’s evaluation scores. Often, areas of the colon look similar to polyps due to lighting and contrast, leading to false bounding boxes and decreasing the mAP and F1-scores. The user can control the number of FPs by adjusting the probability threshold for discarding bounding boxes. A large threshold will reduce the number of FPs and increase the amount of missed polyps and vice versa. Therefore, our model tolerates more FPs and minimizes the amount of missed polyps in clinical practice. We discuss this point further in the following subsection.

Furthermore, our evaluation shows a significant advantage in using REPP and RT-REPP to reduce the number of FPs. In many cases, the FPs increase when using REPP or RT-REPP, e.g., in video 9 or 1 in Table 10. This happens if a false detection is highly significant. For example, the YOLOv5 architecture predicts a box on a bubble, which does not move and stays inside the frame. In this case, the detection score is high and REPP and RT-REPP include the FP. Nevertheless, REPP and RT-REPP reduce small FPs in several frames. In contrast, the YOLOv5 and Faster R-CNN architecture still include these small FPs. Therefore, in exceptional cases, FP can be increased. Nevertheless, longer-lasting FPs are less distracting than FPs with a short duration which might mislead the endoscopist and therefore increase the withdrawal time of the colonoscopy.

Finally, the usability of our system in a clinical setting depends on the financial cost. The system must operate in real-time during the examination, which a delay-prone server-based solution can not sustain. Therefore, every colonoscopy room needs its own system setup with one or more GPUs. Real-time detection needs both fast processing speed and enough VRAM for the video stream, especially while using RT-REPP. The current GPU with the best performance-to-cost ratio for these requirements is the NVIDIA Geforce RTX 3080, which cost around USD 800 in December 2021. Depending on the size of the clinic, the cost to equip the colonoscopy rooms will easily reach several thousand dollars. However, new GPUs are constantly developed, making current GPUs less expensive.

### 4.2. Clinical Use

A big advantage of our system is that it is already fully implemented as a complete package instead of having several conceptual parts. As described before, the system fits right between the video stream from an endoscopy camera, processes the input and displays the image on the clinical monitor. The direct video stream can still be displayed without our processing on a second monitor. Due to our multi-threaded implementation, the processed image is displayed essentially latency-free, which is a must in the clinical setting. Additionally, due to this implementation, in the future slower, more computationally heavy models can be used without having the disadvantage of higher latency. The system is also applicable to the most commonly used endoscopy processors, expecting a resolution of 1920 × 1080 pixels. Hence, the system can be set up easily in any common clinical setting.

As mentioned above, FPs are a topic of discussion for evaluation metrics in the context of clinical practice. An ideal model would only produce TPs, however a real trained model can not. In a clinical setting, false negatives are more dangerous to the patient than FPs. A FP box displayed by the model can be checked by the examiner and determined to be false, whereas a missed polyp may turn out to be fatal for the patient. As such, while common metrics essentially weight FPs and false negatives the same, clinical practice requires an increased weighting on false negatives in order to properly assess the models performance. We optimised the threshold value for the detection of polyps to rather show more FPs than missing a polyp. Nevertheless, the RT-REPP architecture is still achieving high precision values while also selecting a lower threshold. Therefore, our model does produce FPs rather than false negatives. Still, the amount of FPs is limited and does not disrupt the clinical workflow excessively. Nevertheless, the system has yet not been tested in a clinical trial. Therefore, we are planning to execute a clinical trial with the polyp-detection system.

Our code is open source and, as such, any information engineer can compile and install all necessary components by themselves. However, not every clinic has the necessary resources for this task. While remote support is possible in some cases, as of now, our dedicated software engineer needs to visit each clinic personally to solve more serious problems and to install software updates. We are working on a solution to make updates more dynamic and installable for any clinical environment.

## 5. Conclusions

In this study, we have implemented and tested a fully assembled real-time polyp-detection system that can be used directly in clinical applications. For this cause, we have developed and tested an object detection system, the core of our application, which consists of YOLOv5, an object detection CNN, and our novel post-processing step RT-REPP, a modified version REPP [13] for real-time detection. The system was tested on a public benchmark (CVC-VideoClinicDB) and our own newly collected and annotated dataset (EndoData) and surpassed state-of-the-art detectors with an F1-score of 90.25% one the CVC-VideoClinicDB data while still maintaining real-time speed.

Furthermore, we introduced a new performance metric “first detection time”, which measures the time between the first appearance of a polyp and the time of the first detection by the system. We discussed why the trade-off of a higher number of FPs in return for a better recall is more important for clinical application and, hence, why this metric is closer to measuring model performance in clinical application.

We have explained and discussed how our full system is assembled and implemented. The direct advantages are the flexibility derived from open-source installation and the out-of-the-box application placed between the endoscopy video stream and the clinic monitor for an almost latency-free bounding box detection display. While logistic disadvantages remain, such as the need for on-site visits for maintenance, we are working on finding solutions for these issues.

## Figures and Tables

**Table 1:** Overview of polyp detection models on still image datasets. The table includes the following abbreviation: DenseNet-UDCS: densely connected neural network with unbalanced discriminant and category sensitive constraints; ADGAN: attribute-decomposed generative adversarial networks; CenterNet: center network; SSD: single shot detector; YOLO: you only look once; R-CNN: region-based convolutional neural network.

Author	Year	Method	Test Dataset	F1-Score	Speed
Yuan et al. [25]	2020	DenseNet-UDCS	Custom	81.83%	N/A
Liu et al. [26]	2020	ADGAN	Custom	72.96%	N/A
Wang et al. [27]	2019	CenterNet	CVC-ClinicDB	97.88%	52 FPS
Liu et al. [28]	2019	SSD	CVC-ClinicDB	78.9%	30 FPS
Zhang et al. [29]	2019	SSD	ETIS-Larib	69.8	24 FPS
Zheng et al. [30]	2018	YOLO	ETIS-Larib	75.7%	16 FPS
Mo et al. [31]	2018	Faster R-CNN	CVC-ClinicDB	91.7%	17 FPS

**Table 2:** Overview of polyp detection models on video datasets.

Author	Year	Method	Test Dataset	F1-Score	Speed
Nogueira et al. [53]	2022	YOLOv3	Custom	88.10%	30 FPS
Xu et al. [54]	2021	CNN + SSIM	CVC-VideoClinicDB	75.86%	N/A
Livovsky et al. [50]	2021	RetinaNet	Custom	N/A	30 FPS
Misawa et al. [11]	2021	YOLOv3	SUN-Colonoscopy	87.05%	30 FPS
Qadir et al. [55]	2020	Faster R-CNN	CVC-VideoClinicDB	84.44%	15 FPS
		SSD	CVC-VideoClinicDB	71.82%	33 FPS
Yuan et al. [25]	2020	DenseNet-UDCS	Custom	81.83%	N/A
Zhang et al. [40]	2019	SSD-GPNet	Custom	84.24%	50 FPS
Misawa et al. [52]	2019	3D-CNN	Custom	N/A	N/A
Itoh et al. [51]	2019	3D-ResNet	Custom	N/A	N/A
Shin et al. [33]	2018	Inception ResNet	ASU-Mayo-Video-DB	86.9%	2.5 FPS
Yuan et al. [24]	2017	AlexNet	ASU-Mayo-Video-DB	N/A	N/A
Tajbakhsh et al. [23]	2016	AlexNet	Custom	N/A	N/A

**Table 3:** Results of the 5-fold cross-validation for selecting the final model deep learning model. Values displayed in bold font indicate the highest or most optimal results. The abbreviation “adv.” is an acronym for the term “advanced”.

	Precision	Recall	F1	mAP	Speed	Parameter
Faster R-CNN [32]	81.79	85.58	83.64	79.43	15	91 M
YOLOv3 [35]	80.45	82.46	81.44	81.92	41	65 M
YOLOv4 [79]	83.04	83.68	82.36	83.54	**47**	81 M
YOLOv5 (adv.)	**88.02**	**89.38**	**88.70**	**86.44**	43	79 M
SSD [36]	75.52	76.19	75.85	78.69	30	**64 M**

**Table 4:** Prototype components.

Component	Type	Info
CPU	AMD Ryzen 7 3800X	8 Cores, 3.9 GHz
GPU	MSI GeForce RTX 3080 Ti	12 GB GDDR6X
RAM	G.Skill RipJaws V DDR4-3200	2 × 8 GB
Disk	Samsung SSD 970 EVO Plus	500 GB
Mainboard	B550 Vision D	-
Frame Grabber	DeckLink Mini Recorder 4 K	-

**Table 5:** A 5000 frames system test. This table shows the speed of the detection system of two GPUs. Considering an image input with a speed of 50 FPS.

GPU	AI Exe. Count	AI Avg. Exe. Time	AI Evaluation Rate
RTX 3080 Ti	2996	19.5 ms	29.4 FPS
GTX 1050 Ti	313	306.7 ms	3.1 FPS

**Table 6:** Evaluation CVC-VideoClinicDB dataset. This table compares six different polyp detection approaches on the benchmarking data CVC-VideoClinicDB. The first two models are baseline models, and the third is the best model of the current literature. The last three models are different stages of our polyp-detection system. Precision, Recall, F1, and mAP are given in %, and the speed is given in FPS. Values displayed in bold font indicate the highest or most optimal results. The abbreviation “adv.” is an acronym for the term “advanced”.

	Precision	Recall	F1	mAP	Speed	RT Capable
YOLOv5 (base)	92.15	69.98	79.55	73.21	44	yes
Faster R-CNN	93.84	74.79	83.24	79.78	15	no
Qadir et al. [55]	87.51	81.58	84.44	-	15	no
YOLOv5 (adv.)	98.53	76.44	86.09	77.99	**44**	yes
REPP	**99.71**	**87.05**	**92.95**	**86.98**	42	no
RT-REPP	99.06	82.86	90.24	83.15	43	yes

**Table 7:** Detailed detection approaches on the benchmarking data CVC-VideoClinicDB. The first two models are baseline models, and the last three are different stages of our polyp-detection system. F1, and mAP are given in %. The abbreviation “adv.” is an acronym for the term “advanced”.

Video	YOLOv5 (Base)		F-RCNN		YOLOv5 (Adv.)		REPP		RT-REPP	
	**mAP**	**F1**	**mAP**	**F1**	**mAP**	**F1**	**mAP**	**F1**	**mAP**	**F1**
1	78.22	87.41	92.56	88.14	85.17	91.47	94.56	97.44	89.38	94.18
2	87.35	91.87	89.48	89.19	94.62	96.91	97.48	98.48	96.48	97.96
3	75.58	80.09	81.48	77.71	80.18	84.42	86.48	87.64	82.65	85.01
4	90.04	92.16	93.35	90.39	98.00	98.99	98.35	99.50	98.29	98.99
5	76.29	82.53	78.01	85.85	78.40	87.64	83.01	90.71	78.88	88.27
6	86.23	88.59	87.05	89.42	90.07	94.83	92.05	95.43	88.41	92.83
7	60.75	67.15	69.56	78.38	66.23	76.15	74.56	85.71	71.95	82.35
8	53.93	69.52	77.22	82.65	59.16	73.66	82.22	90.11	82.22	90.11
9	74.27	77.29	84.10	87.21	76.50	87.01	89.10	94.18	85.15	91.89
10	75.28	77.36	86.33	86.00	78.22	87.25	91.33	95.29	86.61	92.61
11	90.17	92.19	94.19	94.92	95.41	97.44	99.19	99.50	98.65	99.50
12	30.81	46.22	42.51	60.09	36.78	54.01	47.51	64.86	39.85	57.14
13	84.48	89.48	84.68	87.06	89.37	94.29	89.68	93.83	90.00	94.74
14	74.35	80.49	82.20	86.42	79.09	87.88	87.20	93.05	82.20	90.11
15	48.88	62.62	52.51	66.56	52.18	69.04	57.51	73.15	55.65	71.79
16	89.45	92.97	93.63	90.32	94.54	97.44	98.63	99.50	98.36	98.99
17	52.25	64.61	56.29	68.15	57.77	72.59	61.29	75.78	49.80	65.75
Mean	73.21	79.55	79.78	83.24	77.99	86.09	86.98	92.95	83.15	90.24

**Table 8:** Details of the EndoData. This table shows the details of our own evaluation data (EndoData). Width and height state the size of the used frames.

Video	1	2	3	4	5	6	7	8	9	10
Frames	14,947	18,026	1960	1923	9277	14,362	347	4627	6639	766
Polyps	2	5	1	1	2	5	1	2	4	1
Width	1920	1920	1920	1920	1920	1920	1920	1920	1920	1920
Height	1080	1080	1080	1080	1080	1080	1080	1080	1080	1080

**Table 9:** Evaluation of EndoData. This table compares five different polyp detection approaches on our EndoData dataset. The first two models are baseline models. The last three models are different stages of our polyp-detection system. F1, and mAP are given in %. Values displayed in bold font indicate the highest or most optimal results. The abbreviation “adv.” is an acronym for the term “advanced”.

	Precision	Recall	F1	mAP	Speed	RT Capable
YOLOv5 (base)	78.39	80.54	79.45	77.09	44	yes
Faster R-CNN	81.85	86.20	83.97	81.74	15	no
YOLOv5 (adv.)	86.21	86.43	86.32	82.28	**44**	yes
REPP	**90.63**	**89.32**	**89.97**	**87.24**	42	no
RT-REPP	88.11	87.83	87.97	84.29	43	yes

**Table 10:** Time to first detect on our own dataset (EndoData). This table compares five different polyp detection approaches on EndoData with our new metric time to first detection (FDT). The first two models are baseline models, and the last three are different stages of our polyp-detection system. FDT is measured in seconds. FP denotes the number of FPs in the video. Values displayed in bold font indicate the highest or most optimal results. The abbreviation “adv.” is an acronym for the term “advanced”.

Video	YOLOv5 (Base)		F-RCNN		YOLOv5 (Adv.)		REPP		RT-REPP	
	**FDT**	**FP**	**FDT**	**FP**	**FDT**	**FP**	**FDT**	**FP**	**FDT**	**FP**
1	0.07	201	0.00	159	0.00	155	0.00	109	0.00	150
2	0.68	13	0.62	11	0.51	4	0.51	8	0.51	5
3	0.10	21	0.00	17	0.00	30	0.00	12	0.00	13
4	0.00	234	0.00	198	0.00	145	0.00	135	0.00	123
5	1.33	663	1.07	572	0.93	425	0.93	379	0.93	352
6	0.13	35	0.07	31	0.03	127	0.03	22	0.03	68
7	5.00	50	3.40	33	2.60	51	2.67	22	2.63	28
8	0.20	99	0.08	83	0.05	152	0.05	58	0.05	50
9	0.68	41	0.32	35	0.32	83	0.32	25	0.32	115
10	0.03	22	0.00	19	0.00	15	0.00	13	0.00	9
Mean	0.82	137.9	0.56	118.7	**0.44**	113.5	0.45	**78.3**	**0.44**	91.3

**Table 11:** False positive rate (FPR) on our own dataset (EndoData). This table extentends Table 10 by providing the FPR for five different polyp detection approaches on EndoData. The first two models are baseline models, and the last three are different stages of our polyp-detection system. Values displayed in bold font indicate the highest or most optimal results. The abbreviation “adv.” is an acronym for the term “advanced”. The FPR is given in %.

Video	YOLOv5 (Base)	F-RCNN	YOLOv5 (Adv.)	REPP	RT-REPP
1	88.15	90.39	90.60	93.20	90.88
2	99.28	99.39	99.78	99.56	99.72
3	90.32	92.02	86.73	94.23	93.78
4	45.11	49.27	57.01	58.75	60.99
5	58.32	61.86	68.58	71.00	72.49
6	97.62	97.89	91.88	98.49	95.48
7	40.97	51.26	40.49	61.20	55.34
8	82.37	84.79	75.27	88.86	90.25
9	94.18	94.99	88.89	96.37	85.24
10	77.69	80.13	83.62	85.49	89.49
Mean	77.40	80.20	78.29	**84.72**	83.37

**Table 12:** Detailed evaluation of EndoData. This table shows a comparison of five different polyp-detection approaches on the our EndoData dataset. The first two models are baseline models, and the last three models are different stages of our polyp-detection system. F1 and mAP are given in %, and the speed is given in FPS. The abbreviation “adv.” is an acronym for the term “advanced”.

Video	YOLOv5 (Base)		F-RCNN		YOLOv5 (Adv.)		REPP		RT-REPP	
	**mAP**	**F1**	**mAP**	**F1**	**mAP**	**F1**	**mAP**	**F1**	**mAP**	**F1**
1	72.77	72.69	84.23	82.26	79.25	82.23	89.84	89.43	82.98	84.26
2	86.30	86.71	86.04	90.51	89.06	94.18	92.83	95.91	90.01	94.74
3	85.65	85.71	93.10	92.88	91.20	91.50	99.10	97.99	98.51	97.00
4	70.57	73.88	82.88	78.17	76.96	79.99	85.43	85.36	83.67	83.99
5	39.45	54.84	44.23	56.79	45.84	58.98	49.60	63.98	49.28	62.40
6	90.22	90.94	94.02	92.11	96.13	96.00	98.38	97.48	96.75	97.50
7	15.12	34.89	29.13	47.81	21.66	43.40	31.72	53.33	28.41	46.39
8	91.14	86.35	96.32	92.71	96.66	94.43	99.46	98.48	98.67	97.00
9	77.49	80.87	78.48	84.72	82.61	87.44	85.11	89.29	81.61	86.59
10	88.73	87.08	88.28	89.10	91.95	94.43	95.82	96.50	92.28	94.91
Mean	77.09	79.45	81.74	83.97	82.28	86.32	87.24	89.97	84.29	87.97

## Data Availability

The first dataset used for the analysis of this article is available in the GIANA challenge repository (https://endovissub2017-giana.grand-challenge.org/, accessed on 18 December 2022). The second dataset (EndoData) used during the analysis is available from the corresponding author on reasonable request.

## Abbreviations

The following abbreviations are used in this manuscript:

CRC	Colorectal cancer
CNN	Convolutional neural network
CAD	Computer-aided detection
CADx	Computer-aided diagnosis
SSD	Single-shot detector
REPP	Robust and efficient post-processing
RT-REPP	Real-time robust and efficient post-processing
COCO	Common objects in context
JSON	JavaScript object notation
YOLO	You only look once
YOLOv5	You only look once (version 5)
FDT	First detection time
AI	Artificial intelligence
GIANA	Gastrointestinal image analysis
WCE	Wireless capsule endoscopy
CEM	Context enhancement module
GAN	Generative adversarial network
FastCat	Fast colonoscopy annotation tool
FPS	Frames per second
GPU	Graphical processing unit
R-CNN	Region based convolutional neural network
SSIM	Structural similarity
ResNet	Residual neural network
SVM	Support vector machine
CSPNet	Cross stage partial network
SPP	Spatial pyramid pooling
VGG-16	Visual Geometry Group-16
FPN	Feature pyramid network
PANet	Path aggregation network
IoU	Intersection over union
